# Ab initio molecular dynamics free energy study of enhanced copper (II) dimerization on mineral surfaces

**DOI:** 10.1038/s42004-022-00688-2

**Published:** 2022-06-28

**Authors:** Kevin Leung, Jeffery A. Greathouse

**Affiliations:** Sandia National Laboratories, MS 1415, Albuquerque, NM USA

**Keywords:** Geochemistry, Density functional theory

## Abstract

Understanding the adsorption of isolated metal cations from water on to mineral surfaces is critical for toxic waste retention and cleanup in the environment. Heterogeneous nucleation of metal oxyhydroxides and other minerals on material surfaces is key to crystal growth and dissolution. The link connecting these two areas, namely cation dimerization and polymerization, is far less understood. In this work we apply ab initio molecular dynamics calculations to examine the coordination structure of hydroxide-bridged Cu(II) dimers, and the free energy changes associated with Cu(II) dimerization on silica surfaces. The dimer dissociation pathway involves sequential breaking of two Cu^2+^-OH^−^ bonds, yielding three local minima in the free energy profiles associated with 0-2 OH^−^ bridges between the metal cations, and requires the design of a (to our knowledge) novel reaction coordinate for the simulations. Cu(II) adsorbed on silica surfaces are found to exhibit stronger tendency towards dimerization than when residing in water. Cluster-plus-implicit-solvent methods yield incorrect trends if OH^−^ hydration is not correctly depicted. The predicted free energy landscapes are consistent with fast equilibrium times (seconds) among adsorbed structures, and favor Cu^2+^ dimer formation on silica surfaces over monomer adsorption.

## Abstract (web summary)

Heterogeneous nucleation of metal oxyhydroxides on mineral surfaces is key to environmentally relevant crystal growth and dissolution, but the cation dimerization steps remain largely unexplored. Here, the authors use ab initio molecular dynamics calculations to examine the coordination structure of hydroxide-bridged Cu(II) dimers, and the free energy changes associated with Cu(II) dimerization on silica surfaces.

## Introduction

Adsorption and trapping of metal cations on to material and mineral surfaces at low ion coverages are key geochemical topics that are much studied for mineral growth and dissolution^1,2^, ion exchange^3^, toxic ion remediation^4^, particle attachment^5^, harvesting Li or actinides from sea water^6^, and other applications related to critical elements^7–11^. The surface density and speciation of adsorbed metal cations depend on the material surface chemistry, binding sites, electric double layers, pH, and salt concentrations. At high metal concentrations and/or high pH, metal hydroxides or other mineral salts precipitate via heterogeneous nucleation, which is a major physical chemistry research topic^12–16^.

At intermediate surface coverage, divalent and trivalent metal cation clusters (“dimers”) and polymeric species have been demonstrated to adsorb on mineral surfaces, clay edges, and in confined aqueous media using X-ray absorption fine structure (XAFS) spectroscopy measurements^4,17–25^. The surface concentration of dimers strongly affects cation uptake, especially as pH varies^23^. Dimers also exist in aqueous solution at sufficiently high pH and metal concentration, and in the presence of organic ligands^26–28^. This important intermediate, pre-nucleation regime, bridging single ion adsorption and precipitation phenomena, is far less studied. Some pertinent, urgent mechanistic questions include whether dimers or ionic aggregates directly form on the surface, or first form in water and then adsorb; what the dimer adsorption configurations are; and whether aggregates made up of multiple highly charged cations, strongly bound to hydroxo (OH^−^) and/or oxo (O^2−^) bridges and to the surface, can reversibly desorb and achieve equilibrium configurations in experimental time scales.

Focusing on divalent and multivalent cation dimer species, the electrostatic repulsion between cations is overcome by OH^−^ and/or O^2−^ bridges, plus the surrounding water/high dielectric media. Al^3+^ dimers, which can be bridged reversibly by either two OH^−^ or a single O^2−^, have arguably received the most fundamental science studies due to their relevance to nuclear waste at high pH conditions^29–34^. In recent years divalent transition metal cation dimers and trivalent lanthanide dimers have become areas of technological significance^6,17,18^. Cluster-based density functional theory (DFT) calculations, with implicit solvent approximations, have been applied to predict favorable Ln^3+^ dimerization tendencies in nanopores^18^. Confinement in nanopores is predicted to enhance lanthanide dimerization, in qualitative agreement with experiments. However, DFT appears to overestimate the magnitude of lanthanide metal cation dimer binding free energies relative to the monomers^18^. Furthermore, these DFT cluster calculations account for surface and nanoconfinement effects on lanthanide dimerization by tuning the dielectric constant, which has to be approximated since it varies with distance from the surface. Obtaining good agreement between DFT/cluster free energy predictions and experimental findings for the Al^3+^ dimer has also been challenging^32^. This suggests that the DFT cluster method, a widely used tool, may need to be re-calibrated for metal cation dimerization phenomena.

This work focuses on Cu^2+^ dimers and is motivated by previous XAFS experiments and classical force field-based molecular dynamics studies^4^. We apply ab initio molecular dynamics (AIMD) and free energy calculations to examine Cu^2+^ dimer adsorption on silica surfaces, and compare the structural and energetic properties of adsorbed dimers with those in aqueous solutions. Unlike DFT cluster calculations, the AIMD simulation cell used herein permits explicit treatment of the silica substrate. Unlike classical MD simulations, AIMD trajectories, which are propagated using DFT-predicted forces, permit picosecond time scale proton transfer via the Grotthuss mechanism and can equilibrate different protonation states; therefore dimer formation, dissociation, and Cu^2+^ desorption from the silica surface can occur in concert with proton transfer as needed^3,35^.

XAFS analysis involves the use of model systems for structural assignment. Our predicted structures, therefore, provide structure refinement and motifs that can be used to guide future XAFS analysis. Another main focus of this work is to compare dimerization free energies on silica surfaces with those in pure water. AIMD, in conjunction with potential-of-mean-force (PMF) methods which rigorously calculate free energy changes along a reaction coordinate, has been successfully applied to estimate the acidity constants of mineral surfaces and to compare the free energies of desorption of different metal cations^36–42^. Here we extend these methods to dimerization reactions.

**Fig. 1:**
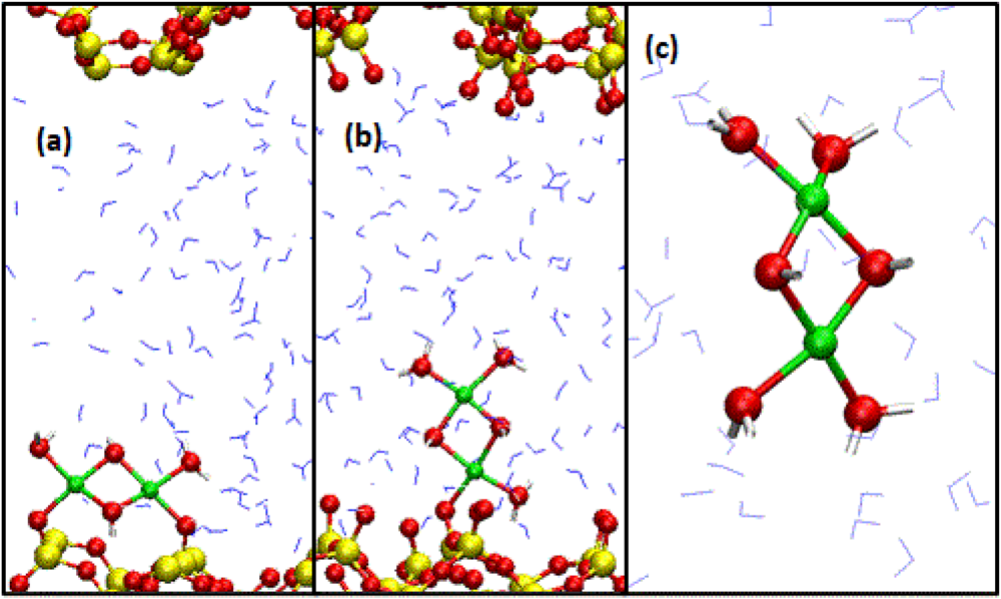
Types of dimers. **a**, **b** Two types of Cu^2+^ dimers on silica surfaces. **c** Cu^2+^ dimer in water. Yellow, red, blue, white, and green represent Si, O, O(water), H, and Cu atoms, respectively. Only silica atoms and the H_2_O/OH^−^ O-atoms directly coordinated to Cu^2+^ are depicted as spheres; otherwise, they are lines or rods.

We use a reconstructed silica model to examine a dimer with both Cu^2+^ cations initially adsorbed on nearby SiO^−^ surface sites (Fig. 1a); and a vertical Cu^2+^ dimer with only one of the Cu^2+^ bound to the surface (Fig. 1b). A dimer in water (Fig. 1c) is also considered for comparison. All these dimers have two bridging OH^−^ groups linking the two Cu^2+^. As will be discussed, it is non-trivial to find a “reaction coordinate” that controls AIMD/PMF calculations, which adds to the challenge of modeling metal cation dimers. We also conduct DFT cluster calculations to compare with our AIMD predictions and elucidate the reason that previous cluster calculations appear to overestimate dimerization tendencies^18^. While quantitative comparison with experiments is challenging due to a lack of atomic length-scale experimental structure at most water-material interfaces, and due to the difficulty in determining the pH in some AIMD simulation cells, our predicted dimerization free energy landscapes help elucidate key mechanisms governing cation dimerization processes on mineral surfaces.

## Results

### Cu^2+^ coordination structures

Figure 1 depicts the three systems examined in this work. In Fig. 1a, a Cu^2+^ dimer with bridging OH^−^ groups lies roughly parallel to the surface, with each Cu^2+^ initially coordinated to one surface SiO^−^ (i.e., “monodentate”). Upon equilibration one of the Cu^2+^ is detached from the surface but the dimer remains horizontal. In Fig. 1b, the dimer is oriented perpendicular to the surface along the Cu-Cu axis. Initially, the Cu closer to silica is coordinated to two surface SiO^−^ groups (“bidentate”), but upon conducting AIMD simulations, the surface complex becomes monodentate. Vertically oriented, monodentate adsorbed Cu^2+^ dimers also form spontaneously in classical MD simulations^4^. Finally, for comparison, we also consider a Cu^2+^ dimer in liquid water. The last simulation cell has a net +2${{{{{\rm{|}}}}}}e{{{{{\rm{|}}}}}}$ charge, and a standard background compensating charge is included; simulation cells containing silica are charge-neutral.

**Fig. 2:**
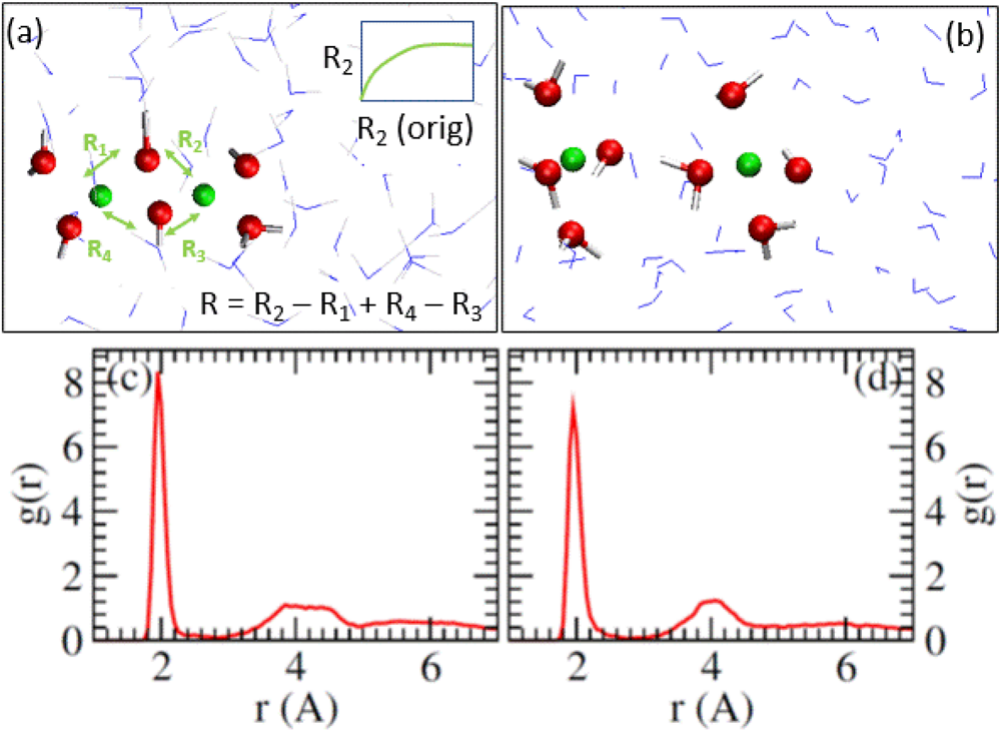
Structural predictions. **a** AIMD snapshot of Cu^2+^ dimer in pure water. **b** The dimer is mostly dissociated, with a H_3_${\rm O}_{2}^{-}$ bridge linking the two Cu^2+^. **c**, **d** ${g}_{{{{{{\rm{Cu}}}}}}-{{{{\rm{O}}}}}}(r)$ between the Cu and O sites for panels (**a**, **b**), respectively. In panel (**a**), the reaction coordinate $R$ is also illustrated; the schematic in the insert indicates the renormalization of a Cu-O distance ${R}_{2{{{{{\rm{orig}}}}}}}$ into ${R}_{2}$ which is an integral part of $R$. A constraint is applied for panels (**b**, **d**); hence those results should be considered qualitative.

First, we consider the structural properties of Cu^2+^ dimers. The average Cu-Cu distances in all Fig. 1 configurations are 2.89–2.91 Å, in reasonable agreement with the $\sim$2.95 Å value reported in a majority of XAFS measurements on different mineral surfaces or clay edges^4,20–24^, but disagree with the $\sim$2.65 Å value reported in some experimental studies^19,25^. Figure 2a depicts an equilibrated AIMD snapshot of a Cu^2+^ dimer in pure water. Each Cu^2+^ is coordinated to 4 equatorial H_2_O or OH^−^ in a square-planar geometry; all the Cu^2+^ and O atoms highlighted are coplanar on average. This structure has been suggested in the majority of XAFS interpretations^4,19–25^. Unlike Al^3+^ dimers in high pH conditions^30^, we do not observe oxo (Cu-O^2−^-Cu) bridges.

Figure 2c depicts the AIMD pair correlation function ${g}_{{{{{{\rm{Cu}}}}}}-{{{{\rm{O}}}}}}(r)$ between both Cu^2+^ and O atoms in H_2_O or OH^−^ in water. The first minimum is at a distance of *r* = 2.85 Å. Integrating to this cutoff yields hydration numbers of ${N}_{w}$ = 4.2 and 4.4 for the two chemically equivalent Cu^2+^, averaging to 4.3. The small discrepancy is a measure of statistical uncertainty. Since the Cu^2+^ share two OH^−^ bridges, the dimer has a total ${N}_{w}$ = 6.6. Similar $g(r)$ are obtained for Cu^2+^ dimers adsorbed on silica surfaces, except that the second peaks have more structure (not shown); the mean coordination numbers for Fig. 1a, b, where the Cu dimer is adsorbed on silica surfaces, are 4.15 and 4.17 per Cu^2+^, respectively. In solid state materials, the formation of bimetallic Cu^2+^ complexes bridged by organic ligands is well known in the literature^26^. In these paddlewheel structures, the coordination environment about each Cu^2+^ consists of four atoms (O atoms as in copper(II) acetate^27^ in a square plane. Full octahedral coordination geometry is achieved with a coordinated solvent molecule on one side and the other Cu^2+^ on the other side. Typical Cu-Cu distances in these compounds are approximately 2.6 Å denoting covalent bonding, significantly smaller than the Cu-Cu distances we predict in aqueous media, and only slightly longer than the bond length of 2.56 Å in metallic copper^28^.) Cu-dimers bridged by OH^−^ or oxo groups are also found in active catalytic sites in zeolites^43–45^.

To further shed light on the Cu^2+^ hydration structure, we also consider nearly dissociated Cu^2+^. Figure 2b depicts a configuration taken from the dissociation end-point of the hydrated dimer. Each Cu^2+^ is coordinated to a OH^−^. The corresponding ${g}_{{{{{{\rm{Cu}}}}}}-{{{{\rm{O}}}}}}(r)$ is shown in Fig. 2d; it is similar to that for the undissociated dimer (Fig. 2c). ${N}_{w}$ = 4.1 and 4.5, averaging 4.3 per Cu^2+^. The reason for the asymmetry between the two Cu^2+^ is apparent from Fig. 2b; the OH^−^ group coordinated to one Cu^2+^ is part of a (H${}_{3}$O${}_{2}$)^−^ bridge between the two cations, while the other Cu^2+^ has an unshared OH^−^. Unlike Fig. 2a, c, the AIMD trajectory used for Fig. 2b, d has a constraint of ${A}_{o}{(R-{R}_{o})}^{2}/2$, ${A}_{o}$ = 4 eV Å^2^ and ${R}_{o}$ = 4.1 Å, to maintain the partially dissociated structure. This latter system does not represent an equilibrium condition, and the ${g}_{{{{{{\rm{Cu}}}}}}-{{{{\rm{O}}}}}}(r)$ there is meant as a qualitative guide.

Some XAFS interpretations of adsorbed Cu^2+^ include approximately one axial H_2_O at $\sim$2.73 Å from the Cu^2+^, with substantial statistical uncertainty about the axial coordination (e.g., ${N}_{w}$(axial) = 1.2$\pm$0.6)^4^. This would correspond to a square pyramidal or distorted octahedral coordination sphere with dynamic distortion. Our AIMD simulations on either the hydrated dimer or the dissociated dimer reveal little or no sign of axial Cu-O coordination centered around 2.7 Å (Fig. 2c, d).

This discussion highlights a feature specific to copper. Cu^2+^ is an outlier among first-row transition metal ions. AIMD simulations have predicted Cu^2+^(H_2_O)${}_{n}$ complexes, $n$ = 5, due to Jahn-Teller distortion^46,47^, instead of the octahedral $n$ = 6 complexes associated with most other divalent first-row transition metal cations^48^. Experimentally, dynamical fluctuations among ${N}_{w}$ = 4, 5, and 6 have been reported^49^; the AIMD results may be reflections of such variations. In contrast, with a OH^−^ in the first hydration shell, square-planar Cu^2+^(OH^−^)(H_2_O)_3_ with ${N}_{w}$ = 4 has been predicted in AIMD simulations^35^. Reduced hydration numbers as the metal cation effective charge decreases are also predicted and exploited as the reaction coordinates in AIMD simulations of Al^3+^ complexes^5^. Such a hydration shell is also found in cluster-based models with 4–5 explicit H_2_O molecules^50–52^. With 5 explicit H_2_O, a ${N}_{w}$ = 4 configuration which has one H_2_O outside the Cu^2+^ first hydration shell, is favored over the 5-coordinated Cu^2+^ configuration by 0.04 eV. Clusters with two hydration shells of H_2_O molecules stabilize the 5-coordinated Cu^2+^OH^−^ complex over the 4-coordinated complex by a small 0.07 eV^50^. Unlike cluster-based calculations, AIMD simulations explicitly include dynamic water motion in both the first and second hydration shells, and the AIMD-predicted ${N}_{w}$ = 4 should be considered more accurate than cluster results when the same DFT functional is used. As will be discussed, the coordination structure has a significant impact on the choice of the reaction coordinate.

### Cu^2+^ dimerization free energies

The above structural elucidation helps lay the foundation for discussing the standard state dimerization free energy $\Delta {G}_{{{{{{\rm{dimer}}}}}}}$. $\Delta {G}_{{{{{{\rm{dimer}}}}}}}$ is obtained by integrating the free energy profile $\Delta W(R)$ as a function of the reaction coordinate $R$ using the potential-of-mean-force (PMF) method (Sec. S5). A key ingredient is the choice of the scalar reaction coordinate $R$. We attempted two reaction coordinates. The obvious first choice, the distance between the two Cu^2+^ (${R}_{{{{{{\rm{Cu}}}}}}-{{{{{\rm{Cu}}}}}}}$), yields discontinuous $\Delta W({R}_{{{{{{\rm{Cu}}}}}}-{{{{{\rm{Cu}}}}}}})$ when tested on the systems of Fig. 1a, c. The reason is that ${R}_{{{{{{\rm{Cu}}}}}}-{{{{{\rm{Cu}}}}}}}$ is somewhat orthogonal to the initial breaking of one of the two Cu-O bonds needed to dissociate the dimer (Sec. S2, Fig. S2).

In this work, we apply a (to our knowledge) novel, single coordinate of the form $R$=${R}_{1}$–${R}_{2}$+${R}_{3}$−${R}_{4}$ (Fig. 2a, Fig. S4). ${R}_{n}$ are Cu-O distances, but are renormalized so that ${R}_{n}$=${R}_{B}$+$[{R}_{n}({{{{{\rm{o{rig}}}}}}})-{R}_{B}]/\{1+{[{R}_{n}({{{{{\rm{o{rig}}}}}}})-{R}_{A}]}^{4}\}$, with the constants being ${R}_{A}$=2.0 Å, and ${R}_{B}$=4.0 Å. The formula ignores the dimensionality (expressed in Å). The smallest ${R}_{n}({{{{{\rm{orig}}}}}})$ observed is about 2 Å and corresponds to an unbroken Cu-O bond. At such distances ${R}_{n} \sim {R}_{n}({{{{{\rm{orig}}}}}})$, because the denominator reduces to unity. In addition, this transformation assures that, as $R$ increases to break the first Cu-O bond (any of ${R}_{n}$(orig), $n$ = 1, 2, 3, or 4, elongated beyond $\sim$4 Å), the ${R}_{n}$ associated with that Cu-O bond reaches a plateau of $\sim$4 Å, and can no longer affect the reaction coordinate $R$ if ${R}_{n}$(orig) further increases. This allows $R$ to control the breaking of the remaining Cu-O-Cu bridge via a controlled increase of a different Cu-O distance ${R}_{n{\prime} }$(orig). $R$ ranges from $\sim$0.0 Å in an intact dimer to about $\pm$4 Å when both initial Cu-O bridging bonds are broken. The convergence properties are depicted in Sec. S5, Figs. S5, S6 and S7. This coordinate requires designating two special O atoms. However, it does not require the bond-breaking sequence to be determined, and it has the empirical, unforeseen advantage that the two special O atoms always remain bonded to at least one of the Cu^2+^ each during all trajectories. Despite that, our PMF calculation with coordinate $R$ is only piecewise reversible. Care must be exercised when assuming that it is globally reversible (Sec. S6, Figs. S8, S9; see also the concluding section), which would require relabeling of the designated O-atoms.

While $R$ has limitations, we emphasize that there is no guarantee that a better coordinate exists. As an example of coordinates that work for other systems but will not for our system, consider the popular class of reaction coordinates related to ${N}_{w}$, which is agnostic to water molecule identities. For example, the Na^+^/Cl^−^ ion pair in water, the total ${N}_{w}$ of the two ions has proven an effective coordinate^53^. In the case of (Cu^2+^)${}_{2}$, the total ${N}_{w}$ for both Cu^2+^, after eliminating double counting associated with O atoms coordinated to both Cu^2+^, goes from 6 in the dimer to 8 as the cations separate. Unfortunately, as alluded above, one of the undissociated Cu^2+^ dimer can also acquire an axial H_2_O to reach ${N}_{w}$ = 7 at a free energy change of $<$0.1 eV^50^. This free energy cost will prove to be lower than ${{{{{\rm{|}}}}}}\Delta {G}_{{{{{{\rm{dimer}}}}}}}{{{{{\rm{|}}}}}}$. As such, increasing ${N}_{w}$ will only cause the dimer to acquire more coordinating water molecules, rendering ${N}_{w}$ unsuitable as a dimer dissociation coordinate.

Another pragmatic approach is to break two bridging Cu^2+^-(OH^−^) bonds sequentially. Since two-dimensional PMF^53^ is currently too costly for AIMD simulations, this means two separate reaction coordinates and two PMF runs would be needed. After the first Cu-OH^−^-Cu linkage is broken, we also need to avoid the possibility that the OH^−^ will be replaced by another H_2_O molecule diffusing between the two Cu^2+^ and reforming the broken bridge. In heterogeneous situations where the two bridges are non-equivalent, such as dimers adsorbed on mineral surfaces, a sequential approach further requires that the bond-breaking sequence is pre-determined. Therefore this choice has none of the advantages of our ultimate choice $R$, but shares the disadvantage of having to specify special O atoms.

**Fig. 3:**
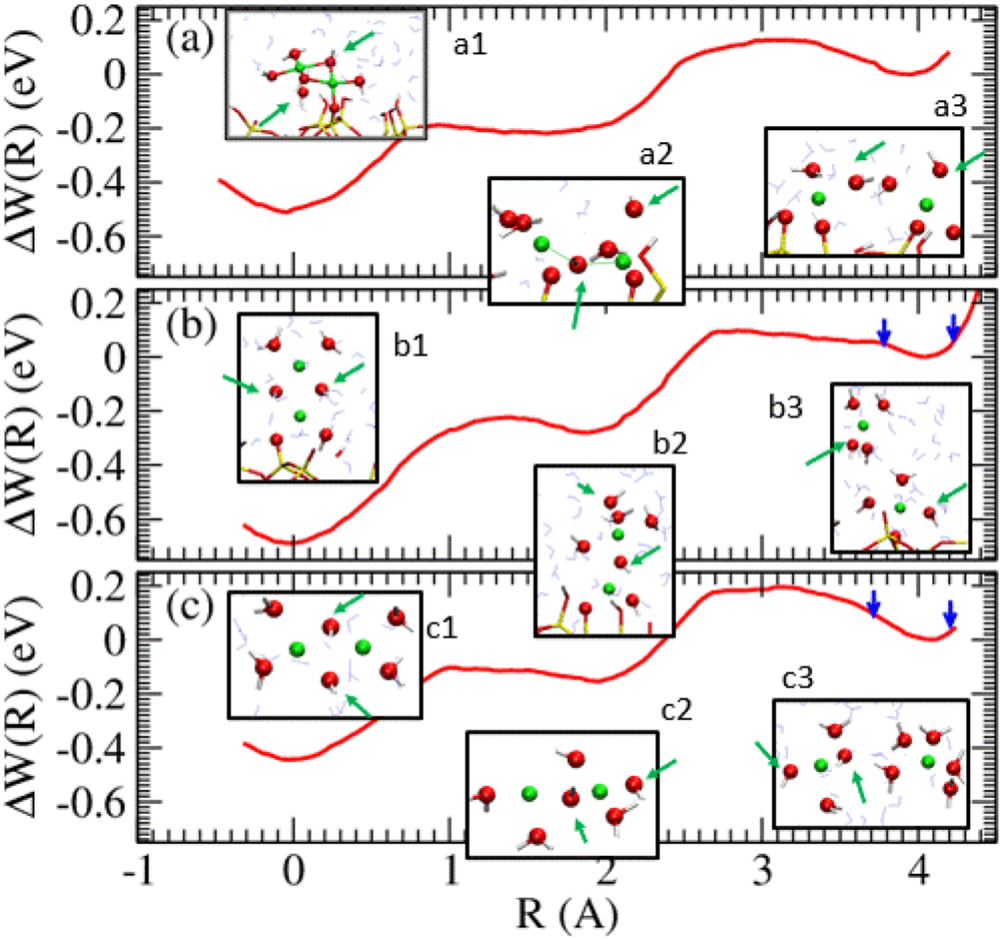
AIMD/PMF profiles along the reaction coordinate *R* for the systems of Fig. 1a–c. AIMD snapshots along the profile are depicted as insets. Green arrows indicate the OH^−^ positions in the snapshots; note that OH^−^ identity can vary in picosecond time scales. The blue arrows represent integration limits. For further detail see Sec. S4. $\Delta {G}_{{{{{{\rm{dimer}}}}}}}$=−0.51, −0.59, and −0.38 eV for (**a**–**c**).

Figure 3a–c depicts $$\Delta W(R)$$, computed using this reaction coordinate for Fig. 1a–c, respectively. The $$\Delta W(R)$$ are qualitatively similar. The most stable minima at $$R \sim$$0.0 Å, (inset panels a1, b1, and c1), the middle minima/inflection points (insets a2, b2, and c2), and the outer minima (insets a3, b3, and c3), approximately correspond to breaking zero, one and two Cu-O-Cu linkages, respectively. After an initial Cu^2+^-O bond is broken, a chain of (H_2_O)$${}_{q}$$ molecules, $$q > $$1 (with one of these molecules possibly deprotonated, i.e., becoming a OH^−^), can always be identified as linking the two metal ions. On silica surfaces, the most stable Cu dimer free energy wells (panels a1 and b1) are more stable by 0.50–0.66 eV relative to the outermost minimum, while a more shallow well of 0.44 eV is observed in pure water (c1).

The outermost minimum cannot extend beyond $R \sim$4 Å by the construction of $R$. However, in Sec. S1, Fig. S1, we show that releasing all constraints after reaching $R$ = 4.0 Å in Figs. 1b, 3b system leads to spontaneous Cu^2+^-dimer dissociation, in the sense that the two Cu^2+^ are found to diffuse almost freely with respect to each other. This suggests that the outermost minimum in $\Delta W(R)$ is already at the dimer dissociation limit. The snapshot associated with the deepest minimum in Fig. 3b exhibits monodentate adsorption of the Cu^2+^ dimer on the silica surface after equilibration, despite the fact that we initiate this configuration as bidentate. As mentioned above, monodentate configurations are in agreement with experiments and CMD simulations^4^. Although both Cu^2+^ ions in Fig. 1a, b are coordinated to the silica surface via a single Cu^2+^-SiO^−^ bond, they should be considered distinct due to their different Cu-Cu axis directions. As dissociation proceeds, proton transfers occur in the Cu^2+^ hydration shells via the Grotthuss mechanism, with some OH^−^ transforming into H_2_O and vice versa. This is depicted in more detail in Sec. S3, Fig. S3.

To calculate the dimer association free energy $\Delta {G}_{{{{{{\rm{dimer}}}}}}}$, we integrate the $\Delta W(R)$ profiles (Sec. S4, Table S1). In the case of Fig. 1a, the outer minimum corresponds to Cu^2+^ adsorbed at different binding sites, and is used as the reference dissociated state. (Note that this reference state should not be identified with twice the adsorption free energies of an isolated Cu^2+^, calculated in our previous work^35^, because the two Cu^2+^ are in reasonably close proximity and repel each other.) In contrast, the reference states associated with Fig. 1b, c should be consistent with the release of a Cu^2+^ to infinity inside the bulk electrolyte, at a fictitious 1.0 M standard state concentration. This entails a translational entropic correction (Sec. S4). With these reference states, $\Delta {G}_{{{{{{\rm{dimer}}}}}}}$ = −0.51$\pm$0.02, −0.59$\pm$0.03, and −0.38$\pm$0.02 eV for the three cases, where the negative sign indicates that dimerization is favorable. The difference in $\Delta {G}_{{{{{{\rm{dimer}}}}}}}$ between Cu-dimer on silica surfaces and Cu-dimer in water has therefore converged to several times the statistical uncertainties.

Note that, to obtain $\Delta {G}_{{{{{{\rm{dimer}}}}}}}$, we integrate over the global or local minima of $\Delta {G}_{{{{{{\rm{d}}}}}}{imer}}$ to capture the volume element (entropy) associated with them. Such integrations should in principle include a determinant that arises from the conversion of the composite reaction coordinate $R$ to the Cartesian coordinates of the atoms. Given the complexity of our reaction coordintaes, and the expectation that the motion out of each local minimum of $R$ is expected map on to the breaking of a single Cu-O bond, we have omitted the determinant (set it to unity). In effect, we have approximated the integration as though $R$ is locally and linearly dependent on the Cartesian coordinates of two bonded atoms. See Sec. S4 for further discussions. Note that such integrations are seldom discussed or carried out in the literature; free energy differences are instead often reported as the difference between the bottoms of local potential wells^54–56^, which is a more severe approximation than the one used herein.

From the PHREEQC database^57^, it can be inferred that 2 Cu^2+^(OH^−^) $\to$ Cu^2+^_2_(OH^−^)_2_ yields a pH-independent −0.33 eV at standard state conditions, in reasonable agreement with our AIMD PMF predictions for Fig. 3c. However, such an agreement in the absolute value of dimerization free energies may be slightly fortuitous because the DFT/PBE functional used herein does not predict absolute energies with chemical accuracy (see “Methods”); instead, we focus on comparing dimerization in water and on silica surfaces where cancellation of errors should occur. Comparing with the measurements in Knight et al.^4^ is more difficult because the pH is not specified in Fig. 3c. Assuming Fig. 1c is conducted at solution pH equals to pK${}_{a}$ = 8 for a H_2_O coordinated to an isolated Cu^2+^ in water^57^, and using the pH = 6 0.3 mM Cu^2+^ concentration experimental condition^4^, Cu^2+^ dimerization in water is found to yield a non-standard-state $\Delta G$ = +0.07 eV using our AIMD $\Delta {G}_{{{{{{\rm{dimer}}}}}}}$. Therefore dimerization in water is slightly unfavorable under those conditions. In contrast, Cu^2+^ dimerization on silica surfaces would be favored under the same conditions due to the more negative $\Delta {G}_{{{{{{\rm{dim}}}}}{er}}}$ there. These findings are in qualitative agreement with XAFS spectroscopy of Cu^2+^ in silica nanopores which shows more pronounced Cu-Cu backscattering under confinement than on a non-porous material surface^4^.

Comparing Fig. 3b, c, the presence of the surface to which the Cu^2+^ dimer is anchored makes the dimer binding free energy more favorable by −0.21 eV. This is reasonable because of the increased negative surface charges on SiO^−^ groups at the interface, which stabilize the inherent coulombic repulsion within the dimer with a net +2${{{{{\rm{|}}}}}}e{{{{{\rm{|}}}}}}$ charge.

We cannot directly compare the horizontal (Fig. 3a) and vertical (Fig. 3b) $\Delta {G}_{{{{{{\rm{dimer}}}}}}}$ because the dissociated dimer reference states are different. However, from the fact that the Fig. 1a configuration starts out with both Cu^2+^ coordinated to SiO^−^ groups but equilibrates to only one Cu^2+^ attached to the surface, there does not appear to be an energetic advantage for a horizontal Cu^2+^ dimer. We conjecture that the vertical dimer (Fig. 1b), spontaneously observed in CMD simulations, is more favorable than the horizontal dimer (Fig. 1a).

The adsorption of an isolated Cu^2+^(OH^−^) to this silica surface model has been predicted to exhibit a standard-state $\Delta {G}_{{{{{{\rm{ads}}}}}}}$ = −0.47 eV using similar AIMD PMF methods^35^. which is less favorable than attaching a Cu^2+^(OH^−^) to a Cu^2+^ on the silica surface in a vertical configuration (−0.59 eV, Fig. 1b). However, at the initial stages of flow experiments^4^, the surface concentration of Cu^2+^ should be low. Entropic effects should favor adsorption of Cu^2+^ monomers on to unoccupied silica surface sites, until the Cu^2+^ surface concentration is built up and adsorbed Cu^2+^ dimers become favorable. Since Cu^2+^ desorption costs ($-\Delta {G}_{{{{{{\rm{ads}}}}}}} \sim$ 0.47–0.59 eV) are modest, and only low overall barriers exist (0.1–0.2 eV, Fig. 3), the use of standard kinetic equations with Arrhenius dependence on free energy barriers would suggest that reversible monomeric and dimeric Cu^2+^ adsorption can occur in at most a 20 s time scales.

### DFT cluster calculations for comparison

Another attribute of silica surfaces is that they provide negatively charged SiO^−^ surface groups that stabilize the positively charged dimer. To isolate the role of the net charge in the vicinity of the dimers, we apply the more computationally economical Gaussian suite of programs^58^ to examine Cu^2+^ dimer complexes, using the polarizable continuum model (PCM)^59^ implicit solvent with ${\varepsilon }_{o}=78$ to treat the aqueous environment outside the cluster model. We consider

1 $${{{{{\rm{Cu}}}}}}^{2+}({{{{{{\rm{H}}}}}}}_{2}{{{{{\rm{O}}}}}})_{4-m}({{{{{\rm{OH}}}}}}^{-})_{m}+{{{{{\rm{Cu}}}}}}^{2+}({{{{{{\rm{H}}}}}}}_{2}{{{{{\rm{O}}}}}})_{4-n}({{{{{\rm{OH}}}}}}^{-})_{n}\\ \quad\to ({{{{{\rm{C}}}}}}{u}^{2+})_{2}({{{{{{\rm{H}}}}}}}_{2}{{{{{\rm{O}}}}}})_{p}({{{{{\rm{OH}}}}}}^{-})_{m+n}+(8-m-n-p){{{{{{\rm{H}}}}}}}_{2}{{{{{\rm{O}}}}}},$$

where all H_2_O and OH^−^ species are understood to be in the Cu^2+^ first hydration shells.

The reference states are two isolated Cu^2+^(H_2_O)_3_(OH^−^) ($m$ or *n* = 1) and/or Cu^2+^(H_2_O)_2_(OH^−^)_2_ ($m$ or $n$ = 2) at 1.0 M concentration to maintain mass balance and charge neutrality. As $m+n$ increases from 2 to 3 to 4, the net charge on the Cu-complex decreases from +2${{{{{\rm{|}}}}}}e{{{{{\rm{|}}}}}}$ to +1${{{{{\rm{|}}}}}}e{{{{{\rm{|}}}}}}$ to 0, and the Gaussian-predicted $\Delta {G}_{{{{{{\rm{dimer}}}}}}}^{(g)}$ increases in magnitude from −0.53 eV to −0.76 eV and −0.84 eV, respectively. Larger $n$ correspond to higher electrolyte pH, which is predicted to enhance dimerization. This is reasonable because overall charge neutrality should reduce the electrostatic repulsion that drives the Cu^2+^ apart. $m$ = 1, $n$ = 1, and $p$ = 4 correspond to the AIMD simulations of Fig. 1c.

**Fig. 4:**
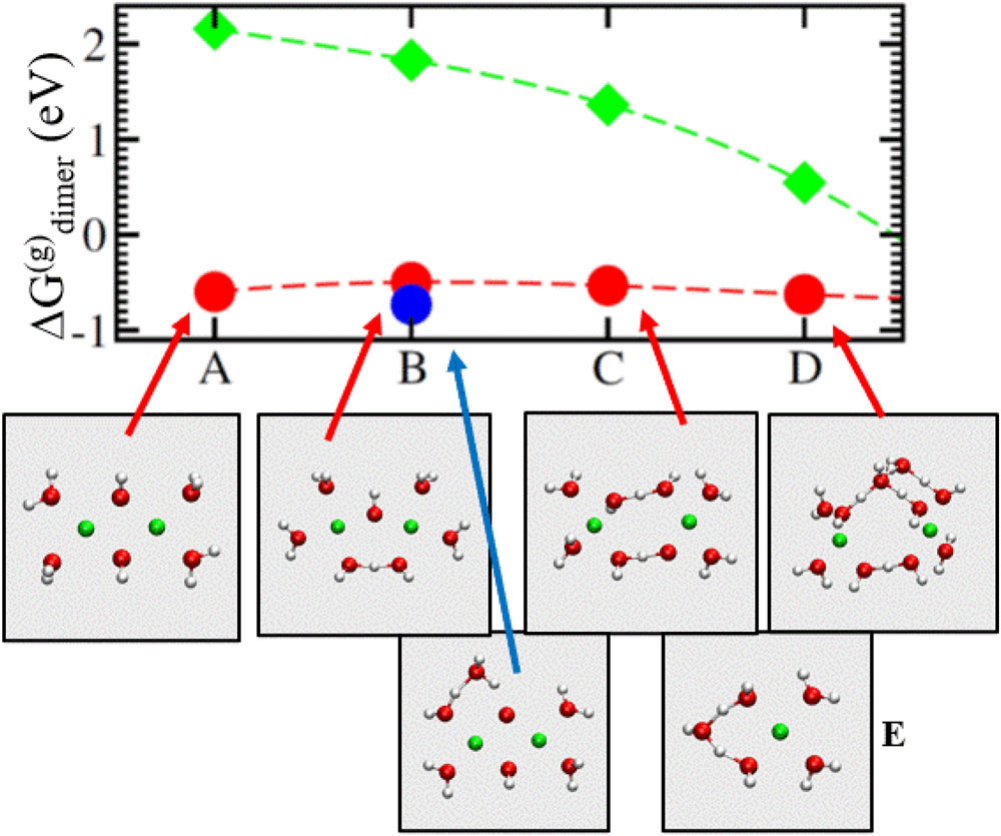
Cluster-based free energy profiles associated with (Cu^2+^)_2_ (OH^−^)_2_ (H_2_O)_p_. Red circles denote cluster dimerization free energies ($\Delta {G}_{{{{{{\rm{dimer}}}}}}}^{(g)}$) associated with structures A–D, where *p* = 4, 5, 6, and 8; implicit PCM solvent is also applied. D is taken from a snapshot of AIMD simulations and further optimized. B’ (blue circle) also has *p* = 5; but one of the H_2_O is coordinated to OH^−^ and neither of the Cu atoms. Green diamonds have configurations similar to those for red circles, but PCM is not used. Dashed lines are guides to the eye.

To show that the dielectric around the cluster also plays a large role, we further turn off PCM, corresponding to a gas phase environment. This yields a $\Delta {G}_{{{{{{\rm{dimer}}}}}}}^{(g)}$ = +2.16 eV repulsive association free energy for $m$=$n$=1 (Fig. 4). Without the outer solvation shells represented by the implicit PCM solvent, the $m$=$n$=1 cluster dissociates. The implicit solvent stabilizes a complex with a net charge of $q$=+2${{{{{\rm{|}}}}}}e{{{{{\rm{|}}}}}}$ over that of two separate complexes with net charges $q$=+${{{{{\rm{|}}}}}}e{{{{{\rm{|}}}}}}$ via the Born formula, $\Delta G \sim$−$(1-1/{\varepsilon }_{o}){q}^{2}/2a$, where $a$ is the effective radii which is only slightly larger for the dimer complex than for the two Cu^2+^ monomers.

While these trends are qualitatively reasonable, the quantitative $\Delta {G}_{{{{{{\rm{dimer}}}}}}}^{(g)}$=−0.53 eV value for (Cu^2+^)_2_(H_2_O)_4_(OH^−^)_2_ disagrees with the $\Delta {G}_{{{{{{\rm{dimer}}}}}}}$=−0.38 eV computed using AIMD, or the similar PHREEQC value of −0.33 eV. In Sec. S6, we include tests to show why AIMD/PMF predictions should be reliable. Even more problematic for the cluster method, as more H_2_O molecules are added to the cluster and the Cu-Cu distance increases the $\Delta {G}_{{{{{{\rm{dimer}}}}}}}^{(g)}$ remains roughly constant or becomes even more exothermic: from −0.53 eV (Fig. 4A) to −0.50 eV (Fig. 4B) to −0.53 eV (Fig. 4C) to −0.62 eV (Fig. 4D), relative to two isolated Cu^2+^(H_2_O)_3_(OH^−^). This is unreasonable because, as the number of intervening H_2_O molecules increases and the two Cu^2+^ become well-separated, the “dimer” should approach two isolated, hydrated Cu^2+^ clusters, and $\Delta {G}_{{{{{{\rm{dimer}}}}}}}^{(g)}$ for such clusters should approach zero, just like in Fig. 3c.

We stress that the exact numbers associated with Fig. 4 quantitatively depend on basis sets, the choice of implicit solvent, and other factors. Nevertheless, our qualitative conclusion that cluster-based calculations with the PCM implicit solvent significantly overestimate dimerization stability is consistent with our previous work^42^. In Sec. S7, we argue that the discrepancy between $\Delta {G}_{{{{{{\rm{dimer}}}}}}}^{(g)}$, and the corresponding AIMD $\Delta {G}_{{{{{{\rm{dimer}}}}}}}$, mainly arises from the PCM error in solvating the OH^−^ species. The PCM cavity radius for OH^−^ may be adjusted to correct this error^59^, but we leave the development of robust cluster-based dimerization free energy calculations to future work.

## Conclusions

Using AIMD simulations, we find that Cu^2+^ dimers are 6-coordinated and bridged by two OH^−^ whether they are in water or adsorbed on silica surfaces. Using AIMD potential-of-mean-force (PMF) calculations with a custom-built reaction coordinate, we find that the Cu^2+^ dimer association free energy ($\Delta {G}_{{{{{{\rm{dimer}}}}}}}$) is a favorable −0.51 to −0.59 eV (Fig. 1a, b) on model silica surfaces, depending on whether a dissociated Cu^2+^ is ejected into the electrolyte or stays on the surface. In liquid water, $\Delta {G}_{{{{{{\rm{dimer}}}}}}}$ is −0.38 eV, in reasonable agreement with the PHREEQC database. This value is up to 0.21 eV less favorable than on silica surfaces. This is partly due to the negative surface charges on the model silica surfaces, and may partly be due to the decreased dielectric response of water at interfaces^18^. These results are relevant to heavy metal ion adsorption, nucleation, and dissolution phenomena. Performing both AIMD and cluster-DFT calculations allows us to compare the respective predictions, and to conclude that cluster-DFT with implicit solvent may overestimate the stability of metal cation dimers if the OH^−^ bridges linking the metals are not sufficiently hydrated by explicit water molecules. Finally, the predicted free energy landscapes reveal that Cu^2+^ cations likely directly dimerize on silica surfaces as opposed to forming in water and adsorbing as such. Dimer dissociation occurs on time scales of seconds and should be reversible under experimental conditions, suggesting these pre-nucleation events should readily reach equilibrium. It would be of significant interest to image such dimerization events^5^.

Computationally, the potential-of-mean-force calculations required to obtain these results have proved challenging and have required a (to our knowledge) new, not completely ideal, complex reaction coordinate. Given the importance of dimerization and polymerization of transition metal ions in geochemistry and other applications, we propose that further research in the computational methods used herein will be highly useful.

## Methods

The Perdew-Burke-Ernezhof (PBE) functional^60^ is applied in all calculations. Finite temperature spin-polarized AIMD simulations apply projector-augmented wave-based Vienna Atomic Simulation Package (VASP)^61–64^, a 400 eV energy cutoff, and $\Gamma$-point sampling of the Brillouin zone. These settings are similar to those in our previous ion desorption work^35,42^. Simulation cells containing silica are charge-neutral, have dimensions 14.2  × 14.2 × 26.0 Å^3^, a Si_40_O_88_${\rm H}_{12}^{4-}$ slab for the reconstructed $\beta$-cristobalite (001) slab, 2 Cu^2+^, and 121 H_2_O molecules. Cells without silica have dimensions 16.0 × 12.0 × 12.0Å^3^, 2 Cu^2+^, and 81 H_2_O, with two of them deprotonated. The Cu pseudopotential used does not include pseudovalent $3p$ electrons. Each PMF is evaluated using umbrella sampling and the scalar reaction coordinate illustrated in Fig. 2. Gaussian calculations apply the lanl2dz basis set for Cu^65^, and the 6-311+G(d,p) basis for O and H atoms. Other details are given in the “Results” section and Sec. S7.

The spin-singlet gas phase (Cu^2+^)_2_(OH^−^)_2_(H_2_O)_4_ cluster is found to be more stable than the triplet cluster by 0.007 eV when using the PBE functional, the VASP code, and a uniform background charge. To within the accuracy of this functional, the two are effectively identical in energy; at the temperature of the AIMD simulations, a mixture of singlet and triplet should be found. The two spin states yield very similar PBE-optimized structures at *T* = 0 K. Experimental and more advanced electronic structure calculations also suggest that the singlet state is more stable by a small amount, on the order of 10 cm^−1^ ($<$0.01 eV). The triplet state electronic structure is generally more robust to converge. For computational convenience, triplet spin states are enforced in all AIMD and g09 calculations.

DFT/PBE calculations are not as accurate as high-level quantum chemistry approaches. In Sec. S8 and Tables S2–S3, we compare the DFT/PBE-predicted bond lengths and angles for a Cu-dimer compound with those predicted using more accurate electronic structure techniques^66^. Further comparison between DFT/PBE energetics and multi-reference, symmetry broken couple-cluster energetics^66^ should be conducted in the future. Finally, the optimized coordinates of cluster-based DFT calculations are documented in Sec. S9, Table S4–S5 and Fig. S10.

## Supplementary information

Supplementary Information

## Data Availability

All relevant data will be available from Kevin Leung (kleung@sandia.gov).

## Code availability

All analysis software will be available from Kevin Leung (kleung@sandia.gov).
