# Supplementary material for: Ab initio molecular dynamics free energy study of enhanced copper (II) dimerization on mineral surfaces
Source: Commun Chem. 2022 Jun 28;5:76. doi: 10.1038/s42004-022-00688-2 (PMC9814296; doi:10.1038/s42004-022-00688-2)
Supplement: Supplementary file 1 — Supplementary Information [file 42004_2022_688_MOESM1_ESM.pdf]

Supporting Information for:

Ab initio molecular dynamics free fnergy study of enhanced copper (II) dimerization on mineral surfaces

Kevin Leung* and Jeffery A. Greathouse
[1]Sandia National Laboratories, Albuquerque, NM 87185, U.S.A.

Contents

S1. Supplementary Notes 1 .... page S2

S2. Supplementary Notes 2 .... page S3

S3. Supplementary Notes 3 .... page S4

S4. Supplementary Notes 4 .... page S5

S5. Supplementary Notes 5 .... page S9

S6. Supplementary Notes 6 .... page S12

S7. Supplementary Notes 7 .... page S14

S8. Supplementary Notes 8 .... page S17

S9. Supplementary Notes 9 .... page S17

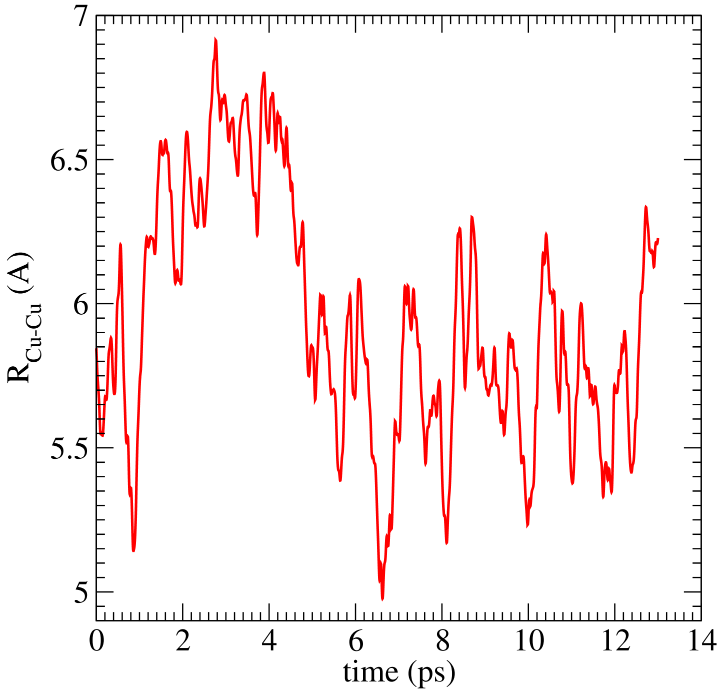

FIG. S1: $R_{\mathrm{Cu-Cu}}$ when the umbrella sampling constraint at the largest R window of the hydrated Cu^{2+} dimer trajectory (Fig. 1c in the main text) is released.

S1. SUPPLEMENTARY NOTES 1

This section discusses the large Cu-Cu distance behavior. Fig. S1 depicts the time evolution of $R_{\mathrm{Cu-Cu}}$ starting from the sampling window with the largest R_o values in Fig. 1b/Fig. 3b of the main text, and releasing the sampling constraint. This simulation is for the Cu-dimer which initially sits vertically on the silica surface. As can be seen, the Cu-Cu distance is 4.9 to 6.9 Å, or 2.0-4.0 Å beyond equilibrium value (2.90 Å) in the bound configuration. It also experiences significant, fast fluctuations. This suggests that the two

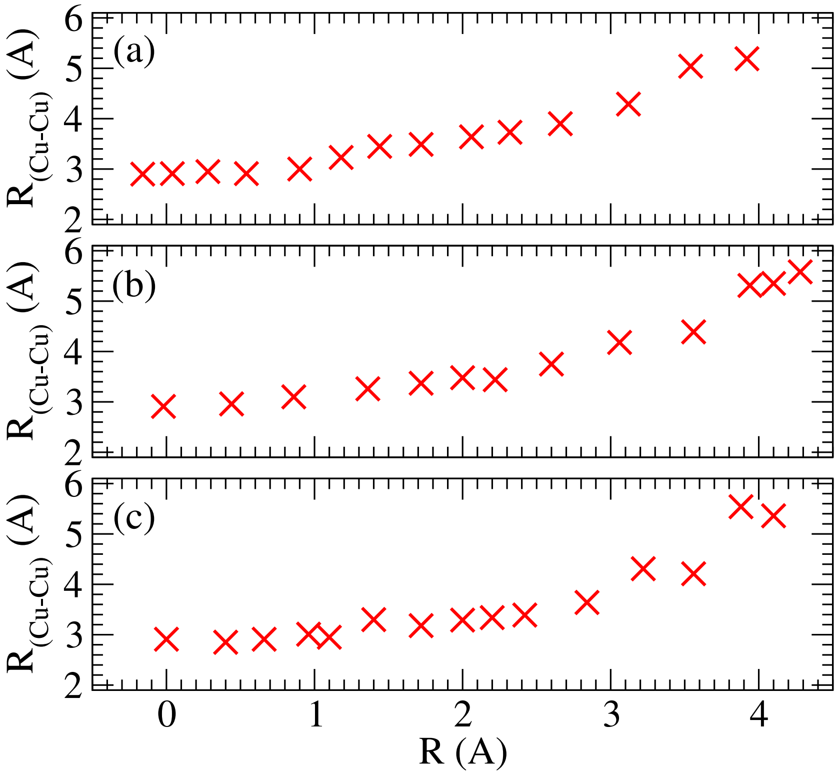

FIG. S2: Correlation between median R in each sampling window and the average $R_{\text{Cu-Cu}}$ in that window. (a)-(c) are for the Cu dimer horizontally and vertically adsorbed on silica surfaces, and in liquid water, respectively; they correspond to the systems of Fig. 1a-c in the main text.

Cu^{2+} are unbound to each other at this stage, and the last constrained R_o window can be considered "infinite separation" for the purpose of calculating free energies.

S2. SUPPLEMENTARY NOTES 2

This section discusses the reaction coordinate vs. the Cu-Cu distance. Fig. S2 depicts the correlation between the median R value in each sampling window, and the mean distance between the two Cu^{2+} cations ($R_{\text{Cu-Cu}}$). Panels a-c correspond to Fig. 1a-c/Fig. 3a-c in the main text. The R<1 Å region, which is almost the entirety of the initial rise in $\Delta W(R)$, is associated with breaking the first Cu^{2+}-O bond and is almost independent of $R_{\text{Cu-Cu}}$. This

explains why our initial attempt at using $R_{\mathrm{Cu-Cu}}$ as the reaction coordinate has resulted in poor control of the dimer dissociation reaction. In contrast, the reaction coordinate R ultimately chosen (Fig. 2a in the main text) is designed to break Cu-O bonds.

Note that the 6-body coordinate R is expected to be most reliable at the initial stages of Cu^{2+} dimer dissociation. This is because at the initial stages ($R \leq 1$ Å), R maps on to the lengthening of one Cu-O bond.

S3. SUPPLEMENTARY NOTES 3

This section discusses the proton transfer behavior. In the pure water simulation (Fig. S3a-c), the two O's marked by black arrows are the two designated Os in the reaction coordinate R. Initially both are OH^- groups bridging the two Cu atoms (Fig. S33a). As dissociation proceeds, one of the Cu-O bond breaks, and the O atom on that broken bond acquires an extra proton from a H_2O further away, becoming an H_2O (Fig. S3b; the OH^- in each snapshot are marked by red arrows). As dissociation further proceeds and two Cu-O bonds associated with the designated O atoms are broken, the second designated OH^- acquires a second proton, becoming a H_2O, while the first designated OH^- loses one of is two protons and turns back into an OH^- (Fig. S3c) As can be seen, two of the H_2O molecules initially coordinated to one of the Cus have diffused away. However, neither of the O designated in the reaction coordinate R is ever observed to diffuse away over the course of the entire AIMD/PMF simulations, except during the reversibility test across more than one windows (Sec. S6, not used in the main text). Note that the panels are rotated with respect to the axes and give the illusion that the Cu's are not constrained in the x- and y-planes.

In the simulation with silica (Fig. S3d-f), significant proton transfer occurs in the Cu coordination shells as well. The red O atoms in panels Fig. S3d-f always indicate those originally coordinated to the Cu atoms before Cu dissociation occurs, unlike in Fig. S3a-c.

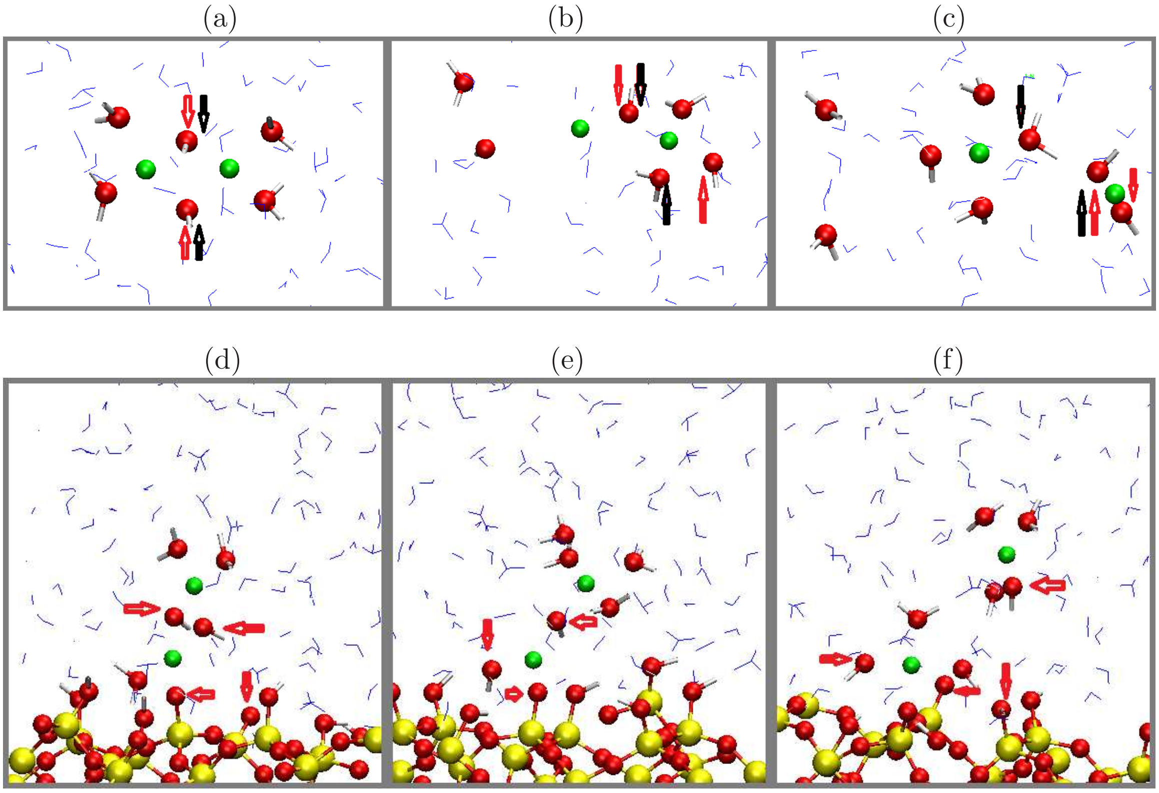

FIG. S3: (a)-(c) Expanded depiction of the three dimer snapshots in Fig. 3c of the main text. Cu, O, and H atoms are in green, blue, and white. Red spheres represent H_2O or OH^- O atoms which were initially coordinated to either of the Cu at the beginning of the AIMD/PMF simulations. (d)-(f) Expanded depiction of the three dimer snapshots in Fig. 3b of the main text. Si, Cu, O, and H atoms are in yellow, green, blue/red, and white. Here, unlike in (a)-(c), red spheres represent H_2O or OH^- O atoms which are coordinated to either of the Cu in the snapshot. Black arrows in (a)-(c) arrows the two O atoms which are part of reaction coordinate R. Red arrows in all panels indicate OH^- in the snapshot.

S4. SUPPLEMENTARY NOTES 4

This section discusses the extraction of $\Delta G_{\rm dimer}$ from $\Delta W(R)$. $\Delta G_{\rm dimer}$ is related to $\Delta W(R)$ via the expression

$$\Delta G_{\rm dimer} = -k_{\rm B}T \log\{\int dR\Omega(R) \exp[\Delta W(R)/k_{\rm B}T]/V_{\rm ref}\}, \tag{S1}$$

where the one-dimensional integral is over an appropriate 3-dimensional, R-dependent volume element $\Omega(R)$ dR, and $V_{\rm ref}$ is the spatial volume associated with the standard state (e.g.,

that of a solute at 1.0 M concentration, which is partly associated with the translational entropy of cation desorption or dimer dissociation).

Details of the AIMD trajectories used to calculate the potential of mean force are given in Table S1. The total trajectory lengths associated with the three configurations in Fig. 1a-c of the main text, excluding equilibration times, are 357 ps, 302 ps, and 456 ps, respectively.

In the Cu-dimer in pure water simulations (Fig. 1c of the main text), the two Cu are constained to have the same x and y coordinates; only their z coordinates differ. The reason is as follows. The constraint in pure water forces the two Cu to be colinear. It prevents rotation of the Cu-Cu bond about the z-axis and allows the use of a simulation cell with smaller x- and y-dimensions than would be the case if the dimer were allowed to rotate. If the AIMD trajectory is sufficiently long, and statistical sampling is adequate, and the simulation cell is sufficiently large, there would be no difference with or without the constraint. This is because of the rotational invariance in bulk liquid water. If sampling statistics is limited, the constraint actually helps because the rotational degree of freedom associated with the Cu-dimer no longer needs to be sampled. In contrast, on or near a surface, the free energy of the system is not invariant to Cu-Cu rotation about the dimer axis. Hence constraint cannot be used. In summary, the use of the constraint in bulk aqueous environment is a computational convenience.

It is non-trivial to extract ΔG_{dimer} from $\Delta W(R)$. When the reaction coordinate R directly maps onto a physical coordinate, like the vertical distance z of a single metal ion from the mineral surface in our previous work,[3,4] R=z, and a cylinder with axis along the z-direction can be chosen for Ω. Even in this case, a choice in the radius of the cylinder still needs to be made. In contrast, in a homogeneous aqueous media, a spherical Ω would be rigorously correct. A rigorous determinant that relate $\Omega(R)$ to the Cartesian coordinates of Cu and O atoms can in principle be constructed, but it involves multi-dimensional integrals that are too complex to evaluate at each time step.

For our dimerization problem, which has a highly non-trivial reaction coordinate R (Fig. 2 of the main text), $\Omega(R)$ is difficult to construct rigorously. As shown in Fig. S2, in the small R region of Fig. 3 of the main text, R is almost independent of $R_{\text{Cu}-\text{Cu}}$. Our attempt to reconstruct a $\Delta W(R_{\text{Cu}-\text{Cu}})$ from $\Delta W(R)$ fails because there is almost no volume element associated with $R_{\text{Cu}-\text{Cu}}$ in the initial stage of reaction. In contrast, in the asymptotic region where the two Cu^{2+} are well-separated, $R_{\text{Cu}-\text{Cu}}$ is the correct coordinate to use; it integrates

system	Z_o	A_o	$t_{\rm tot}$	δ	Z_o	A_o	$t_{\rm tot}$	δ
Fig. 1a	+0.12	4.0	18.7	1.4	-0.20	3.0	15.5	4.3
	-0.50	3.0	17.5	4.6	-0.70	4.0	15.9	3.4
	-0.95	4.0	56.2	11.2	-1.20	3.0	18.7	2.6
	-1.50	3.0	20.5	1.1	-1.80	3.0	34.5	1.3
	-2.20	3.0	21.5	2.0	-2.50	3.0	14.8	5.7
	-2.80	3.0	16.8	8.8	-3.15	4.0	37.9	7.4
	-3.50	3.0	37.6	4.6	unc	0.0	18.6	2.9
Fig. 1b	+0.00	0.0	15.0	2.4	+0.60	3.0	17.7	4.2
	+1.00	3.0	15.0	14.9	+1.25	6.0	33.7	9.0
	+1.35	3.0	14.2	4.3	+1.70	3.0	13.7	6.6
	+2.05	3.0	18.9	3.1	+2.40	3.0	19.8	8.9
	+2.75	3.0	18.3	5.0	+2.90	4.0	17.4	2.9
	+3.10	3.0	28.4	12.6	+3.50	3.0	28.5	3.8
	+3.70	4.0	14.1	14.8	+3.90	3.0	34.8	9.0
	+4.20	3.0	15.1	3.3				
Fig. 1c	+0.00	0.0	23.4	4.0	+0.50	3.0	14.4	3.4
	+0.80	3.0	16.8	3.0	+0.95	6.0	83.2	10.1
	+1.10	3.0	16.6	5.6	+1.40	3.0	22.4	0.9
	+1.70	3.0	87.6	8.8	+2.00	3.0	16.5	4.2
	+2.30	3.0	20.2	1.0	+2.60	3.0	20.3	4.9
	+2.90	4.0	58.2	11.7	+3.20	4.0	16.5	5.5
	+3.50	4.0	23.5	6.3	+3.80	4.0	20.2	3.8
	+4.10	4.0	16.5	7.2				

TABLE S1: Details of umbrella sampling AIMD trajectories with constraining potentials $A_o(R - R_o)^2/2$. R_o is in Å, A_o is in eV/Å^2, and $t_{\rm tot}$ is the trajectory length used in sampling statistics in picosecond. δ, in meV, is the estimated uncertainty (one standard deviation) in each window. In the case of Fig. 1a of the main text, the R values reported are actually the negative of what is shown in this table; the minus sign is a only matter of convention.

to a $\Delta W(R)$-adjusted volume that is to be divided by $V_{\rm ref}$ to yield a dimensionless quantity inside the logarithm expression (Eq. S1).

To accommodate these disparate requirements, we use a two-step procedure. (1) We perform one-dimensional integration of Eq. S1 over the inner (smallest R) and outer (largest R) free energy basins. Since the integrand in Eq. S1 exponentially decreases with $\Delta W(R)$ as R is varied from its local minimum, the predictions are insensitive to the precise 1-D R-integration limits. For Fig. 3a of the main text, where the end point finds both Cu^{2+} coordinated to the surface, the difference between these two integrals is the only contribution to $\Delta G_{\rm dimer}$. (2) For Fig. 3b-c of the main text, we further need to relate the outer free energy well to the translational entropy of a detached Cu^{2+} freely diffusing in the aqueous media at 1.0 M concentration. Here we pick the sampling windows, denoted by the blue arrows in Fig. 3 of the main text, record the approximate minimum and maximum $R_{\rm Cu-Cu}$ ($R_{\rm min}$ and $R_{\rm max}$ respectively) in these windows, and add a value of $-k_{\rm B}T \log[(n\pi/3)(R^3_{\rm max} - R^3_{\rm min})/V_{\rm ref}]$, where $V_{\rm ref}$=55.6×29.9 Å^3 is the volume appropriate to a solute at 1.0 M concentration, and n=2 (hemispherical volume) or 4 (spherical volume) for Fig. 3b and Fig. 3c of the main text, respectively. The corrections to Cu^{2+} separation are -0.09 eV and -0.07 eV for those two cases, respectively, favoring dissociation; $\Delta G_{\rm dimer}$ are thus augmented by +0.09 eV and +0.07 eV for these cases.

Some of the choices made herein are arguably somewhat arbitary. The one-dimensional integration of $\Delta W(R)$ over R is motivated by the fact that increasing R corresponding to breaking successive Cu^{2+}-OH^- bonds. Symmetry factors of 4 or 2 may arguably be included in these integrals and correspond to the possible choices of breaking multiple Cu^{2+}-OH^- bonds, but these $k_{\rm B}T\log(2)$ or $k_{\rm B}T\log(4)$ factors are small and are neglected. Recall that the main focus of this work is to demonstrate that Cu^{2+} dimerization preferentially occurs on silica surfaces relative to liquid water. We expect some cancellation of uncertainties when comparing AIMD predictions for the three cases in the main text using the same $\Omega(R)$ definition, and the overall effect on the relative $\Delta G_{\rm dimer}$ with and without silica would benefit from cancellation of uncertainties. The overall systematic uncertainty in $\Delta G_{\rm dimer}$ in this approach is likely less than 0.1 eV, which is smaller than the discrepancy between the AIMD $\Delta G_{\rm dimer}$ reported in the main text and the corresponding g09 predictions.

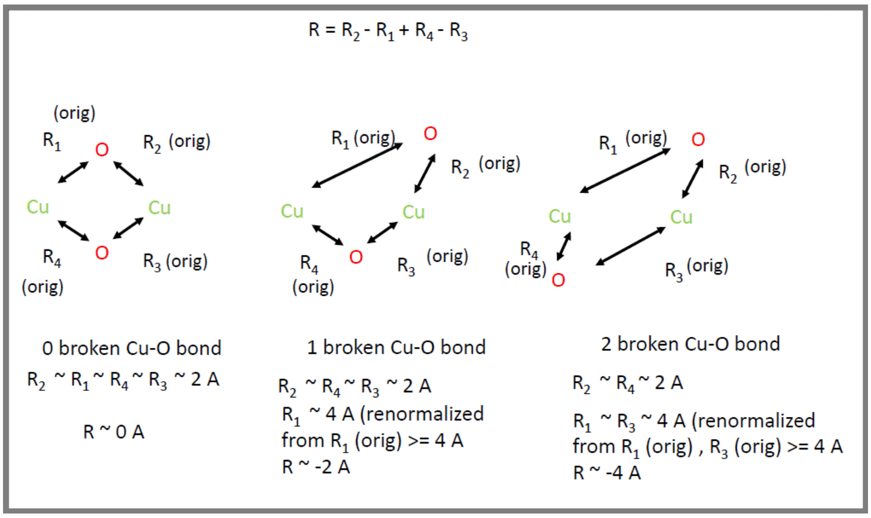

FIG. S4: Schematic of the reaction coordinate R.

S5. SUPPLEMENTARY NOTES 5

This section contains further discussions of the reaction coordinate R. We chose reaction coordinate R, further illustrated in Fig. S4, after rejecting others via trial-and-error, or by examining the energetics associated with adding a water to the Cu hydration shells. An analogy can be made with the early days of AIMD PMF modeling of acid-base reactions. The pKw of liquid water was first computed using a distance constraint.[6] A coordinate constraint approach[7] improved upon it. One of the present authors applied a 4-body coordinate for surfaces. Subsequently, Sprik and coworkers developed and perfected a statistical mechanical approach that annihilates the acid proton from the vicinity of the acid molecule and moves it far into the aqueous region.[8] Thus the later "reaction coordinates" improved upon the earlier ones; without the earlier work the development of the subsequent, improved coordinates might have been much delayed. It is in this spirit that we believe our present work is a useful, pioneering, if imperfect, contribution to computing the free energy of metal cation dimers.

Fig. S5-S7 depict the natural logrithm of the probability ($P(R)$) of the trajectory, already adjusted with $\exp[\beta[A_o(R-R_o)^2/2]$, as a function of R in different sampling windows, and show that these windows fit together.

The bin size is 0.04 A. The green and blue dashed lines are the $\ln[P(R)]$ in each window, truncated such that the unadjusted $P(R)\exp[\beta[-A_o(R-R_o)^2/2]$ probability is at least 0.1%

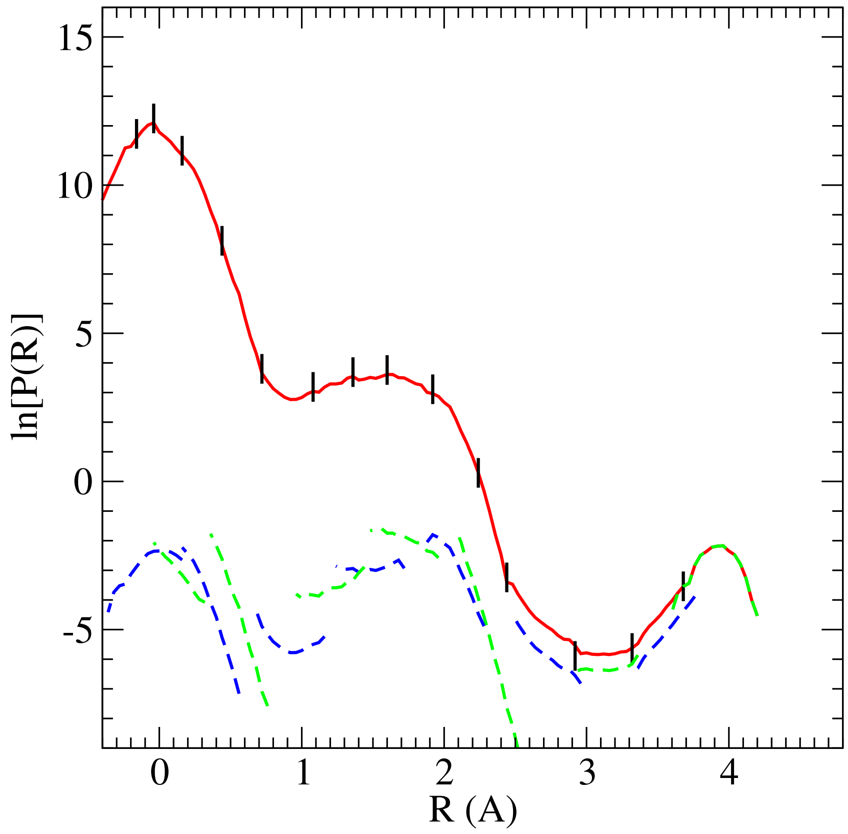

FIG. S5: Details of the $P(R)$ contributions from different sampling windows for Fig. 3a of the main text.

of the total samples (unadjusted probabilities) in the window. Two neighboring windows are usually matched together so that the respective, unadjusted probabilities in their overlapping junction bin are about equal (usually with $P(R)\exp[\beta[A_o(R-R_o)^2/2]>0.01)$. If this leads to an apparent kink in the overall PMF curve, we add a window with R_o in between those two, with a larger A_o, and rerun. Table S1 shows that the Fig. 3b calculation has a few of these added intermediate window. This is because we initially chose R_o in two successive windows which are too far apart and the overlapping bin has small probabilities.

To illustrate some of the choices involved, the inset of Fig. S6 shows that two initial choices of adjacent windows exhibit discontinuous slopes in $P(R)$. The two windows in question had R_o=1.15 Å and A_o=5 eV/Å^2, and R_o=1.35 Å and A_o=3 eV/Å^2, respectively. The original window with R_o=1.15 Å, was run for only 14 picoseconds. A much longer trajectory should

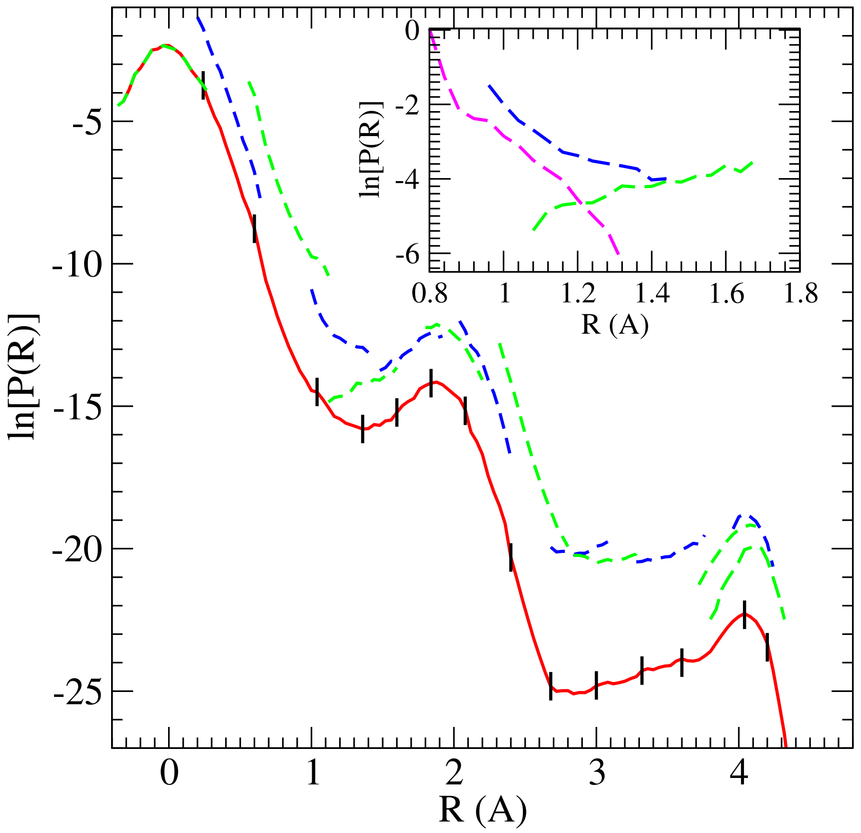

FIG. S6: Details of the $P(R)$ contributions from different sampling windows for Fig. 3b of the main text. In the inset, purple represnets an initial choice of window parameters that yield a significant kink in the $\Delta W(R)$ curve, while blue represents a more converged choice, with statistics collected at a longer time scales; the matching is vastly improved.

improve the matching. We take an alternate approach, taking the configuration end of the R_o=1.35 Å trajectory and restarting another window with R_o=1.25 Å, A_o=6 eV/Å^2 with it, and run this trajectory for a significantly longer time of 33.7 ps after discarding the first picosecond. As can be seen in the inset of Fig. S6, this approach accelerates the convergence of the overall $\Delta W(R)$, smoothing out the apparent kink to a large extent. The change in the overall desorption free energy is minimal compared to the original choice which would require ignoring the kink in the curve; it is only 0.02 eV more favorable for dimerization on silica surfaces. Note that adding an extra window does not always work well. In our previous work,[4] the appearance of a kink is due to the emergence of a second, slow degree

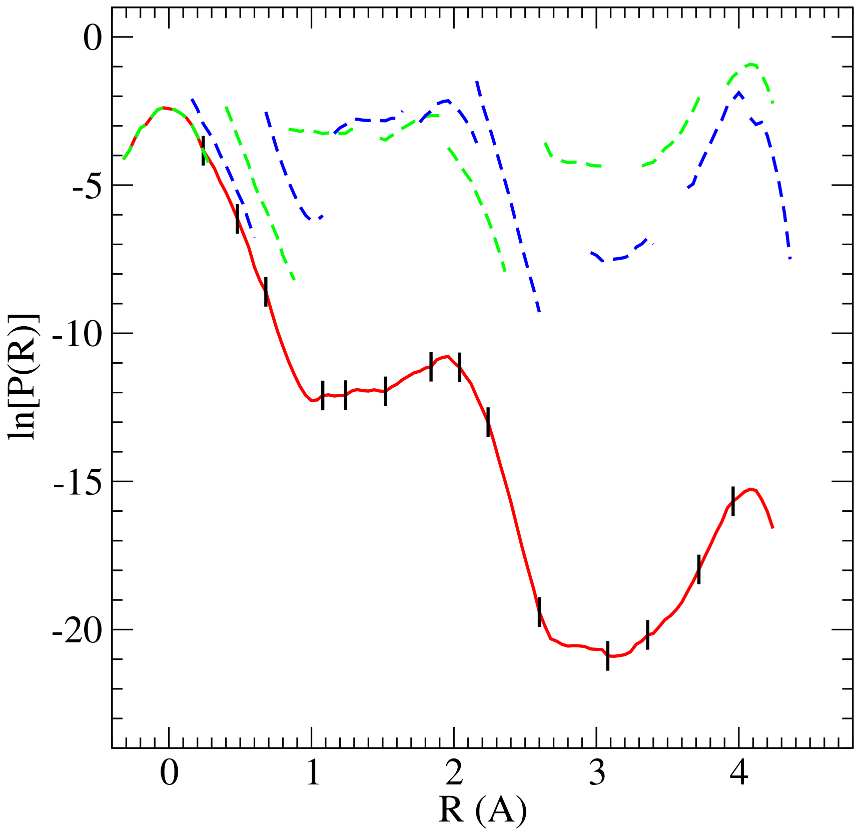

FIG. S7: Details of the $P(R)$ contributions from different sampling windows for Fig. 3c of the main text.

of freedom that requires a secondary (quasi-2D) umbrella sampling.

In summary, we manually but carefully work match the $P(R)$s from different windows.

S6. SUPPLEMENTARY NOTES 6

This section discusses the reversibility of the reaction coordinate R. The $\Delta W(R)$ predictions depicted in Fig. 3 of the main text are obtained by starting from the compact dimer configuration ($R_{\text{Cu-Cu}}$~2.9 Å) and incrementally increasing the constraint R_o value to dissociate the dimer. Here we show that this procedure is piecewise reversible. This demonstration is particularly important for our complex reaction coordinate R, which needs to specify tagged OH^-/H_2O; it is therefore vulnerable to those ions or molecules diffusing

away from the reaction zone, rendering R irrelevant.

Fig. S8a depicts two segments of $\Delta W(R)$ with R_o=1.4 Å, initiated from the R_o=1.1 Å and R_o=1.7 Å windows, respectively. The results are similar. We note that, when we restart from a R_o=2.0 Å window configuration (two R_o windows away) and attempt to recover the R_o=1.4 Å window behavior, that effort fails to recover the original R_o=1.4 Å window $\Delta W(R)$ because one of the bridging OH^- groups that makes up the original R coordinate has acquired a H^+ from the surrounding water, and has diffused away from the bridging position. To recover the $\Delta W(R)$ statistics, we would need to designate a different H_2O or OH^- group as part of R. This suggests that our PMF calculation with coordinate R is piecewise reversible, but care must be exercised when assuming that it is globally reversible. Note that such concerns are not limited to the umbrella sampling method used herein; related approaches like metadynamics face similar reversibility issues.

In the case of the Cu-dimer horizontally adsorbed on silica surfaces (Fig. 1a/3a of the main text), another complication arises. As mentioned in the main text, the initial configuation has each Cu^{2+} coordinated to a SiO^- surface group. With sufficient equilibration in the smallest R_o windows, one of the Cu^{2+} becomes detached from the surface, and is coordinated to H_2O and OH^- only. As the Cu^{2+} dimer starts to dissociate (R>∼0 Å), however, it becomes favorable for both Cu^{2+} to be coordinated to the surface. The AIMD/PMF trajectory lengths used are not always sufficient to "equilibrate" these two configurations (i.e., to allow the multiple occurances of reversible coordination of one of the Cu^{2+} to the surface). Such complications are not infrequently encountered when dealing with the complex energy landscape in AIMD/PMF simulations of water-material interfaces. Fortunately, we find that $\Delta W(R)$ does not strongly depend on whether one or both Cu^{2+} are bound to surface SiO^- groups. Fig. S9a-b depict snapshots of two trajectories at R_o=-0.50 Å, initiated from R_o windows which are smaller and larger, respectively. Throughout their respective AIMD trajectories, one and two Cu^{2+} are coordinated to the silica surface. In other words, full equilibration of the Cu^{2+} configuration has not been achieved. Fortunately, Fig. S8b shows that the $\Delta W(R)$ along these two trajectories are almost identical. Therefore $\Delta W(R)$ is not strongly affected whether one or both Cu^{2+} is/are initially coordinated to the silica surface.

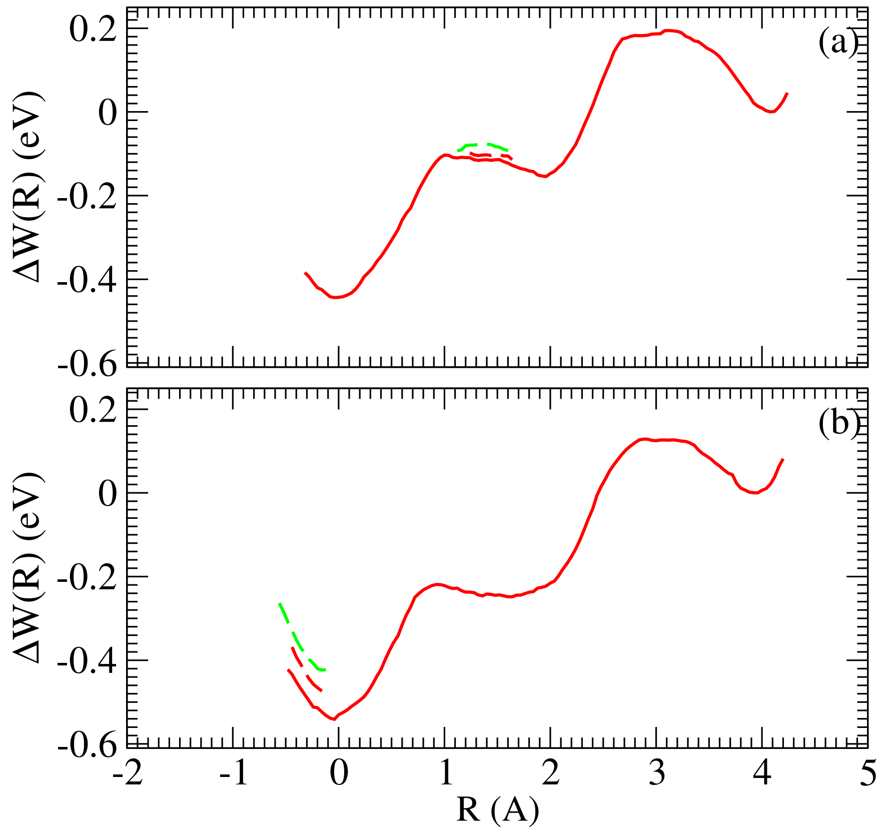

FIG. S8: (a) $\Delta W(R)$ at R_o=1.40 Å; dashed red and green curves are initiated from the R_o=1.10 Å and R_o=1.70 Å windows, respectively. (b) $\Delta W(R)$ at R_o=-0.50 Å. Red and green corressponding to the two snapshots in Fig. S9a-b, respectively.

S7. SUPPLEMENTARY NOTES 7

This section provides more g09 calculation details. We consider general dimerization "reactions" of the form

$$Cu^{2+}(H_2O)_{4-m}(OH^-)_m+Cu^{2+}(H_2O)_{4-n}(OH^-)_n \rightarrow (Cu^{2+})_2(H_2O)_p(OH^-)_{m+n}+(8-p-m-n)H_2O, \tag{S2}$$

where all H_2O and OH^- species are understood to be in the Cu^{2+} first hydration shells. In most cases, m=n=1. The free energies of the reactants and products are computed using the usual harmonic approximations using the g09 package,[1] with thermal and zero point corrections. In addition, to convert from the gas phase (1.0 atm.) pressure reference state

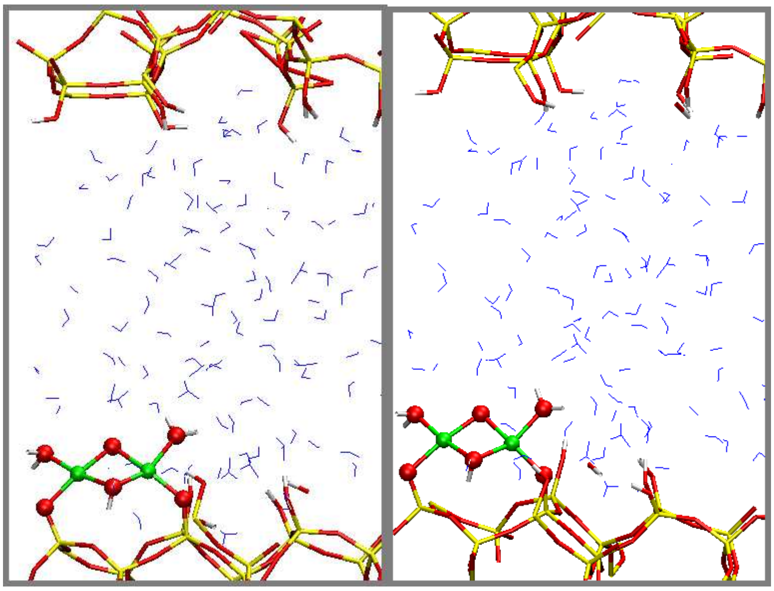

FIG. S9: (a)-(b) Cu^{2+} dimer configurations with one and two Cu^{2+} directly coordinated to SiO^- groups on the silica surface, respectively. Yellow, red, blue, white, and green represent Si, O, O(water), H, and Cu atoms, respectively.

reported by the g09 suite of programs to standard state of 1.0 M Cu^{2+} concentration in water, each Cu^{2+} on the reactant (product) side receives a -0.08 eV (+0.08 eV) correction. H_2O is at 55.6 M concentration, and the correction is -0.19 eV (+0.19 eV).

In the main text, we state that the implicit solvation of OH^- species is the reason the cluster approach (Fig. 4 of the main text) yields free energies which differ from AIMD PMF predictions. Here we provide the rationale. First we consider a single H_2O molecule within the PBE/6-311+G(d,p) cluster/PCM treatment. We ask how accurate the explicit water-implicit water interaction is using this approach. By subtracting the cluster free energy with PCM from that without PCM, the predicted PCM H_2O hydration free energy is $\Delta G_{\rm water}$=-0.27 eV. This is an accurate reflection of the hydration free energy of a H_2O molecule in liquid water,[2] likely by design.

Explicit water-explicit water interactions are also well-represented using the cluster-plus-PCM approach. Thus we next consider

$$2H_2O \rightarrow (H_2O)_2 \tag{S3}$$

where the left side represents two isolated H_2O molecules hydrated by PCM and the right is a water dimer hydrated by PCM. ΔG=-0.03 eV after accounting for entropic corrections

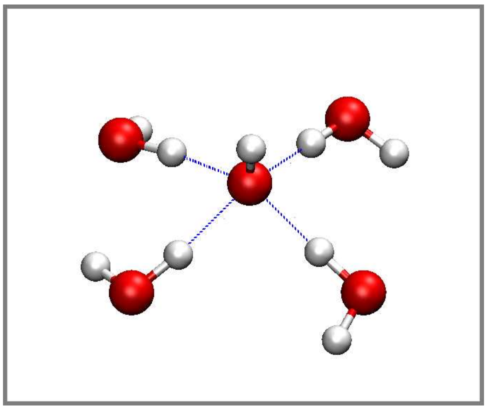

FIG. S10: Optimized $(OH^-)(H_2O)_4$ complex.

discussed above. This near-zero value again shows that PCM is a good approximation of water-water hydrogen bonding.

In contrast, the bare OH^- ion is not accurately "solvated" by PCM. Consider

$$OH^- + 4H_2O \rightarrow (OH^-)(H_2O)_4. \quad \text{(S4)}$$

where all species are PCM-solvated and the cluster on the right side is depicted in Fig. S10. After making the entropic corrections discussed above, the free energy change of the above "reaction" is +0.42 eV, which is significantly different from the ideal 0.0 eV value. This suggests that PCM over-stabilizes the bare OH^- anion. The different configurations of the Cu^{2+} dimer in Fig. 4 of the main text directly exposes its two OH^- to PCM to varying extent, causing errors which are difficult to quantify.

To minimize such PCM errors, we propose that future DFT-cluster calculations have the oxygen site of all OH^- species in the metal cation aggregates to be coordinated with explicit H_2O molecules. Unfortunately, one cannot "optimize" the number of explicit H_2O molecule to be used via a grand canonical scheme within g09/PCM calculations. The reason is that the most energetically favorable case would involve too few H_2O interacting with the OH^-, leaving space for PCM to interact with and overestimate the magnitude of $\Delta G_{\rm dimer}$. Instead, the coordination number of each OH^- can be deduced using AIMD simulations. As mentioned in the main text, the PCM cavity size can also be optimized to yield more accurate results.[5] This manuscript is focused on applying AIMD/PMF methods; we leave such development of cluster calculations to future work.

S8. SUPPLEMENTARY NOTES 8

This section compares the DFT method used in the main text with more accurate methods. Table S2 lists the Cartesian coordinates of "compound 1"[9] predicted using our triplet states DFT/PBE calculations, with the Gaussian suite of programs and a lanl2dz/6-311+G(d,p) basis set. This molecule is studied in Singh *et al.*[9] using more accurate, TPSS functional. Table S3 compares the bond lengths and bond angles predicted using these methods. DFT/PBE overestimates all bond lengths, which it is known to do. It also misses the symmetry-breaking in the bonding environment of the two Cu atoms. We believe the more pertinent issue is the energetics of Cu dimer separation in compound 1; such energy comparisons should be conducted in the future.

S9. SUPPLEMENTARY NOTES 9

This section documents the optimized cluster coordinates.

Atom	x (Å)	y (Å)	z (Å)	Atom	x (Å)	y (Å)	z (Å)
Cu1	1.520073	-0.000055	0.000015	Cu2	-1.520073	0.000054	-0.000016
O1	-0.000021	1.304341	-0.023430	H	0.000032	1.876925	-0.809833
O2	0.000022	-1.304342	0.023426	H	-0.000032	-1.876927	0.809829
N1	3.048249	1.424033	0.006016	N2	3.048414	-1.423932	-0.006035
N3	-3.048413	1.423933	0.006035	N4	-3.048250	-1.424033	-0.006016
C1	3.065770	-1.960682	-1.395763	C2	2.837140	-2.537753	0.953594
C3	4.306129	-0.686566	0.326346	C4	4.305987	0.686812	-0.326591
C5	2.836861	2.538003	-0.953406	C6	3.065656	1.960549	1.395837
C7	-3.065767	1.960682	1.395764	C8	-2.837140	2.537753	-0.953593
C9	-4.306129	0.686567	-0.326343	C10	-4.305987	-0.686811	0.326593
C11	-2.836862	-2.538004	0.953405	C12	-3.065659	-1.960548	-1.395837
H	3.247809	-1.155128	-2.119471	H	3.859324	-2.720700	-1.503956
H	2.091871	-2.418743	-1.612080	H	1.930589	-3.090784	0.675650
H	3.691349	-3.236842	0.925471	H	2.737702	-2.142815	1.974187
H	5.187878	-1.268029	0.002834	H	4.361013	-0.601085	1.421860
H	3.247690	1.154873	2.119411	H	3.859227	2.720533	1.504145
H	2.091769	2.418592	1.612246	H	1.930314	3.090960	-0.675305
H	3.691049	3.237117	-0.925218	H	2.737375	2.143231	-1.974058
H	5.187731	1.268376	-0.003240	H	4.360674	0.601341	-1.422116
H	-2.091773	-2.418592	-1.612248	H	-3.859231	-2.720532	-1.504144
H	-3.247694	-1.154872	-2.119410	H	-2.737375	-2.143232	1.974058
H	-3.691050	-3.237116	0.925218	H	-1.930315	-3.090960	0.675304
H	-4.360673	-0.601340	1.422118	H	-5.187732	-1.268374	0.003243
H	-2.737703	2.142815	-1.974187	H	-3.691348	3.236842	-0.925469
H	-1.930588	3.090784	-0.675650	H	-4.361014	0.601086	-1.421857
H	-5.187878	1.268031	-0.002830	H	-2.091867	2.418742	1.612080
H	-3.859320	2.720700	1.503958	H	-3.247805	1.155128	2.119472

TABLE S2: DFT/PBE-predicted of compound 1 coordinates.[9].

Distance/angle	Singh *et al.*	PBE triplet
Cu1-O3	1.931 Å	2.003 Å
Cu1-O5	1.897 Å	2.003 Å
Cu1-N7	1.996 Å	2.089 Å
Cu1-N8	2.033 Å	2.089 Å
Cu2-O3	1.897 Å	2.003 Å
Cu2-O5	1.931 Å	2.003 Å
Cu2-N9	2.033 Å	2.089 Å
Cu2-N10	1.996 Å	2.089 Å
Cu1-O3-Cu2	101.6^o	113.1^o
Cu1-O5-Cu2	101.6^o	113.1^o
O3-Cu1-O5	78.4^o	81.3^o
O3-Cu1-N7	97.5^o	96.4^o
O5-Cu1-N8	96.3^o	96.4^o
N7-Cu1-N8	87.8^o	86.0^o
O3-Cu2-O5	78.4^o	81.3^o
O3-Cu2-N9	96.3^o	96.4^o
O5-Cu2-N10	97.5^o	96.4^o
N9-Cu2-N10	87.8^o	86.0^o

TABLE S3: Comparison between our distances and angles predicted using our DFT/PBE calculations (triplet), and the method of Ref. 9.

Atom	x (Å)	y (Å)	z (Å)	Atom	x (Å)	y (Å)	z (Å)	Atom	x (Å)	y (Å)	z (Å)
A: O	-2.9654	1.3850	-0.1854	O	-3.0176	-1.3265	0.2189	O	2.9676	-1.3844	-0.1836
O	0.0176	1.2840	-0.1927	O	-0.0172	-1.2843	-0.1988	O	3.0140	1.3306	0.2146
H	-3.8007	1.0653	0.2050	H	-3.0081	-1.8421	1.0471	H	-3.0935	-1.9798	-0.5018
H	3.8035	-1.0629	0.2038	H	0.0328	1.9443	0.5273	H	-0.0328	-1.9491	0.5170
H	3.0080	1.8443	1.0440	H	3.0817	1.9857	-0.5052	H	2.8290	-2.2852	0.1622
H	-2.8252	2.2867	0.1577	Cu	1.4768	-0.0146	-0.0033	Cu	-1.4764	0.0131	-0.0050
B: O	-3.5908	-0.5966	0.5411	O	-2.1814	2.0121	0.1043	O	-1.2072	-1.8689	-0.2028
O	-0.0017	0.5731	-0.7857	O	3.5930	-0.5907	0.5447	O	1.2133	-1.8657	-0.2128
O	2.1785	2.0130	0.1024	H	-4.2722	0.0927	0.6498	H	-2.8702	2.3614	-0.4910
H	-2.3815	2.3571	0.9944	H	-1.5430	-2.3726	0.5614	H	-0.0010	1.5477	-0.6848
H	4.2729	0.1009	0.6471	H	-3.9798	-1.2665	-0.0520	H	-0.0167	-1.9093	-0.1740
H	1.5598	-2.3705	0.5459	H	3.9820	-1.2637	-0.0447	H	2.3760	2.3604	0.9920
H	2.8660	2.3646	-0.4928	Cu	1.7278	0.0092	-0.0552	Cu	-1.7286	0.0110	-0.0545
B1: O	2.8086	1.5955	-0.0275	O	3.2751	-1.1018	0.3888	O	-1.8279	2.8842	0.5699
O	-0.0344	1.0273	-0.4587	O	0.3492	-1.5395	-0.1299	O	-2.9650	0.6612	-0.1380
O	-2.4637	-2.0635	0.4207	H	3.6883	1.4433	-0.4211	H	3.2664	-2.0174	0.0519
H	3.4626	-1.1697	1.3442	H	-0.9309	2.5104	0.4069	H	-0.0422	1.2318	-1.4160
H	0.3977	-2.0787	0.6841	H	-2.6646	1.5954	0.1543	H	-3.3856	0.7575	-1.0122
H	-1.8518	3.1231	1.5126	H	2.4498	2.3968	-0.4517	H	-3.4049	-1.8095	0.4707
H	-2.4216	-2.8431	-0.1643	Cu	-1.3087	-0.4831	-0.1511	Cu	1.5950	-0.0288	-0.0614
C: O	-3.0665	-1.3885	1.2773	O	-1.2516	1.2324	-1.1265	O	1.0869	1.7102	-0.4771
O	3.2262	-1.5553	0.5542	O	-3.2842	1.4970	0.5548	O	3.1234	1.2714	1.3321
O	-1.0703	-1.6216	-0.6723	O	1.3095	-1.1582	-1.2321	H	-3.8922	-1.7933	0.9521
H	-3.2782	-1.0142	2.1528	H	0.0697	1.4747	-0.7873	H	1.4919	2.2151	-1.2064
H	4.1984	-1.4938	0.4983	H	-4.2496	1.4121	0.4402	H	-2.9817	2.1601	-0.1018
H	-1.2893	0.9644	-2.0629	H	3.2965	0.8790	2.2080	H	2.5792	2.0663	1.4908
H	-0.0445	-1.4001	-0.9078	H	-1.0748	-2.3289	-0.0015	H	2.9462	-2.1744	-0.1532
H	1.3593	-0.8428	-2.1526	Cu	2.1596	0.0986	-0.0354	Cu	-2.1499	-0.0993	-0.0354

TABLE S4: DFT/PBE-predicted of optimized coordinates in Fig. 4, with PCM implicit solvent.[9] All distances are in units of Å.

Atom	x (Å)	y (Å)	z (Å)	Atom	x (Å)	y (Å)	z (Å)	Atom	x (Å)	y (Å)	z (Å)
D: O	1.4089	-1.9469	1.2145	O	2.3292	0.9905	1.4586	O	1.2330	-1.6350	-1.4225
O	-1.2152	-1.6046	-1.4597	O	-2.3294	0.8456	1.4321	O	-0.3695	2.5128	1.5486
O	-0.0561	2.5722	-1.1923	O	-1.4950	-1.7872	1.4277	O	2.1543	1.2216	-1.1510
O	-2.0928	0.9618	-1.5156	H	1.6018	-2.7459	0.6832	H	2.8520	1.6472	0.9517
H	2.8831	0.7147	2.2133	H	0.1061	-1.6292	-1.4829	H	-1.5391	-2.5095	-1.2981
H	-3.1402	1.3576	1.2578	H	-0.2229	2.7220	0.5874	H	1.8962	-2.0557	2.0538
H	0.4602	2.0770	1.8292	H	-1.5609	1.5316	1.5420	H	0.0121	3.3819	-1.7269
H	1.5882	-1.4921	-2.3176	H	-0.5237	-1.9407	1.5213	H	-1.7821	-1.3680	2.2628
H	0.8344	2.0663	-1.2838	H	2.5176	0.9958	-2.0259	H	-1.9581	0.4326	-2.3266
H	-1.3178	1.6279	-1.4778	Cu	-1.8451	-0.4150	-0.0562	Cu	1.8712	-0.3388	-0.0709
E: O	1.7610	1.5052	-0.1610	O	-1.0078	1.4546	0.2562	O	1.8349	-1.4202	-0.2695
O	-0.8191	-1.4868	0.2029	H	1.3360	2.3305	-0.4604	H	-1.1053	1.7741	1.1725
H	-1.8977	0.9942	0.0076	H	2.5216	-1.5184	0.4173	H	1.2592	-2.2124	-0.1974
H	-0.9149	-1.7515	1.1371	H	2.4859	1.3290	-0.7885	Cu	0.3982	0.0054	0.0921
O	-2.9790	-0.0771	-0.3368	H	-2.2886	-0.8074	-0.2265	H	-3.2624	-0.0987	-1.2666

TABLE S5: Continuation of DFT/PBE-predicted of optimized coordinates in Fig. 4, with PCM implicit solvent.[9] All distances are in units of Å.

Supplementary References

[1] Gaussian 09, Revision A.1, M.J. Fritsch, *et al.*, Gaussian, Inc., Wallingford CT, 2009.

[2] Varilly, P. & Chandler, D., Water evaporation: a transition path sampling study. *J. Phys. Chem. B* 117, 1419-1428 (2013).

[3] Leung, K,, Criscenti, L.J., Knight, A.W., Ilgen, A.G., Ho, T.A. & Greathouse, J.A. Concerted Metal Cation Desorption and Proton Transfer on Deprotonated Silica Surfaces. *J. Phys. Chem. Lett.*, 9, 5379-5385 (2018).

[4] Leung, K., Ilgen, A.G. & Criscenti, L.J. Interplay of physically different properties leading to challenges in separating lanthanide cations - an ab initio molecular dynamics and experimental study. *Phys. Chem. Chem. Phys.* 23, 5750-5759 (2021).

[5] Long, M.R.P. & Isborn, C.M. Combining Explicit Quantum Solvent with a Polarizable Continuum Model. *J. Phys. Chem. B* 121, 10105-10117 (2017).

[6] Trout, B.L. & Parrinello, M. The Dissociation Mechanism of H_2O in Water by First-Principles Molecular Dynamics. *Chem. Phys. Lett.* 288, 343-347 (1998).

[7] Sprik, M. Computation of the pK of Liquid Water using Coordination Constraints. *Chem. Phys.* 258, 139-150 (2000).

[8] e.g., Churakov, S.V., Labbez, C., Pegado, L. & Sulpizi, M. Intrinsic Acidity of Surface Sites in Calcium Silicate Hydrates and Its Implication to Their Electrokinetic Properties. *J. Phys. Chem. C* 118, 11752-11762 (2014).

[9] Singh, G., Gamboa, S., Orio, M., Pantazis, D.A. & Roemelt, M. Magnetic Exchange Coupling in Cu Dimers Studied with Modern Multireference Methods and Broken-Symmetry Coupled Cluster Theory. *Theor. Chem. Acc.* 140, 139 (2021).
